# An alternate route for cellulose microfibril biosynthesis in plants

**DOI:** 10.1126/sciadv.adr5188

**Published:** 2024-12-13

**Authors:** Eric M. Roberts, Kai Yuan, Arielle M. Chaves, Ethan T. Pierce, Rosalie Cresswell, Ray Dupree, Xiaolan Yu, Richard L. Blanton, Shu-Zon Wu, Magdalena Bezanilla, Paul Dupree, Candace H. Haigler, Alison W. Roberts

**Affiliations:** ^1^ Department of Biology, Rhode Island College, Providence, RI 02908, USA.; ^2^ Department of Biological Sciences, University of Rhode Island, Kingston, RI 02881, USA.; ^3^ Department of Crop and Soil Sciences, North Carolina State University, Raleigh, NC 27695, USA.; ^4^ Department of Physics, University of Warwick, Coventry CV4 7AL, UK.; ^5^ Department of Biochemistry, University of Cambridge, Cambridge CB2 1QW, UK.; ^6^ Department of Plant and Microbial Biology, North Carolina State University, Raleigh, NC 27695, USA.; ^7^ Department of Biological Sciences, Dartmouth College, Hanover, NH 03755, USA.

## Abstract

Similar to cellulose synthases (CESAs), cellulose synthase–like D (CSLD) proteins synthesize β-1,4-glucan in plants. CSLDs are important for tip growth and cytokinesis, but it was unknown whether they form membrane complexes in vivo or produce microfibrillar cellulose. We produced viable CESA-deficient mutants of the moss *Physcomitrium patens* to investigate CSLD function without interfering CESA activity. Microscopy and spectroscopy showed that CESA-deficient mutants synthesize cellulose microfibrils that are indistinguishable from those in vascular plants. Correspondingly, freeze-fracture electron microscopy revealed rosette-shaped particle assemblies in the plasma membrane that are indistinguishable from CESA-containing rosette cellulose synthesis complexes (CSCs). Our data show that proteins other than CESAs, most likely CSLDs, produce cellulose microfibrils in *P. patens* protonemal filaments. The data suggest that the specialized roles of CSLDs in cytokinesis and tip growth are based on differential expression and different interactions with microtubules and possibly Ca^2+^, rather than structural differences in the microfibrils they produce.

## Teaser

Moss mutants lacking CESAs make cellulose microfibrils and have rosette plasma membrane complexes presumed to contain CSLDs.

## INTRODUCTION

The structural integrity of plant cells depends on cellulose, a fibrillar β-1,4-glucan synthesized by mobile integral plasma membrane complexes. In land plants, these cellulose synthesis complexes (CSCs) have a distinctive rosette shape. Available evidence indicates that these CSCs are composed of 18 cellulose synthase (CESA) enzymes and produce a fundamental cellulose microfibril containing 18 glucan chains, although some uncertainty regarding this stoichiometry remains (*1*–*6*). CESAs are required for vascular plant development based on the lethality of *CESA* null mutations (*7*).

Cellulose synthase-like D proteins (CSLDs) also synthesize β-1,4-glucan, raising the possibility of a separate pathway for cellulose microfibril synthesis (*8*). CSLD activity is required to maintain the structural integrity of pollen tubes and root hairs, whose polarized tip growth distinguishes them from other plant cell types. The tips of these cells undergo extensive remodeling of the plasma membrane and deposition of extensible cell wall materials, which must be precisely controlled to enable growth while preventing rupture (*9*). CSLDs also help maintain the integrity of the cell plate, a progenitor structure of the new cell wall that forms during plant cytokinesis (*8*, *10*).

CSCs containing CESAs move in the plasma membrane (*11*), driven by the energy released as glucan chains coalesce to form microfibrils (*12*). In cells that expand by diffuse growth, cortical microtubules guide this movement (*11*) to control microfibril orientation and cell growth polarity (*13*). Catalytically active CSLDs also move in the plasma membrane, but their movements are faster, less linear, shorter in duration, and independent of microtubules (*10*). In vitro, CSLDs formed particles similar in size to CESA trimers. However, no microfibrils were detected in these assays, and it remained unknown whether they form CSC-like complexes or synthesize microfibrillar cellulose in vivo (*8*). Given their roles in polarized tip growth and cytokinesis and their distinct patterns of movement, CSLDs could synthesize microfibrils with distinct properties that facilitate tip growth and cell plate development. However, this has been difficult to investigate experimentally because the products of CESA activity confound in vivo studies of CSLD activity, and CESA and CSLD complexes are unstable in vitro (*1*, *8*).

Unlike vascular plants, mosses have an initial haploid growth phase consisting entirely of tip-growing protonemal filaments that can be propagated indefinitely. The model moss species *Physcomitrium* (formerly *Physcomitrella*) *patens* has CESAs and CSLDs (*14*), and we have shown that CESA activity is required for the transition from tip growth to three-dimensional (3D) diffuse growth required for gametophore formation (*15*, *16*). However, it was unknown whether CESAs are required for protonemal tip growth.

Here, we report that *P. patens* mutants that lack CESAs produce normal protonemal filaments. We also show that CESA-deficient moss lines (i) have plasma membrane rosette structures that are morphologically indistinguishable from CESA-containing CSCs and (ii) synthesize cellulose microfibrils that are structurally indistinguishable from the microfibrils in the primary cell walls of angiosperms.

## RESULTS

### Moss plants lacking CESAs are viable

**Fig. 1.**
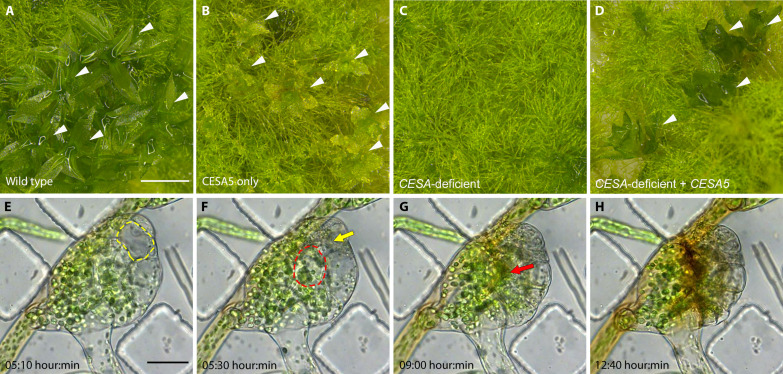
Viable CESA-deficient *P. patens*. (**A**) Wild-type *P. patens* has filamentous protonemata and leafy gametophores (arrowheads). (**B**) Septuple *CESA* knockouts expressing only PpCESA5 have morphologically normal protonemata and stunted gametophores (arrowheads) as described previously (*15*). (**C**) CESA-deficient octuple knockouts are viable with morphologically normal protonemata, but no gametophores. (**D**) Expression of PpCESA5 under the control of the constitutive rice *Actin1* promoter partially rescues gametophore development (arrowheads) in CESA-deficient octuple knockouts. (**E** to **H**) Time-lapse imaging of gametophore buds in CESA-deficient *P. patens* reveals cell rupture [(E) and (F), yellow and red outlines mark fully expanded cells; (F) and (G), yellow and red arrows indicate the positions of the respective cells after rupture] and (H) areas of early senescence marked by accumulation of brown pigment. Scale bar in (A) = 1 mm and applies to (A) to (D). Scale bar in (E) = 50 μm and applies to (E) to (H). Time-lapse interval = 10 min.

Previously, we showed that just one of the eight *P. patens* CESAs, PpCESA5, is sufficient for normal development of both protonemal filaments, which extend by tip growth, and leafy gametophores, which enlarge by diffuse growth (*15*). When we also disabled *PpCESA5* (Phytozome identifier: Pp3c2_13330V3.1; figs. S1 to S5), leafy gametophore development was abolished (Fig. 1, A to D). The CESA-deficient lines produced gametophore buds, but the buds turned brown and stopped growing when they reached about 100 μm in diameter (Fig. 1, E to H). When we investigated the progression of gametophore bud development by time-lapse imaging, we found that the first few divisions followed the documented pattern (*17*) including a transition to 3D growth and rhizoid formation (movies S1 and S2). However, before leaf emergence, interior cells expanded and ruptured as shown in movies S1 and S2 and Fig. 1, E to G, where red and yellow outlines indicate the boundaries of enlarged cells, and arrows indicate the same cells after rupture in later frames. After the second cell rupture, the bud stopped enlarging and accumulated brown pigment (Fig. 1H). The rupturing indicates that CESA activity is required to maintain cell integrity in the early stages of gametophore development. In contrast to gametophore buds, the protonemal filaments of CESA-deficient lines grew vigorously (Fig. 1C) and could be repeatedly subcultured.

To ensure that we had disabled all *P. patens CESA* genes, we verified large deletions in all eight *CESA*s by polymerase chain reaction (PCR) in the CESA-deficient lines (fig. S3). We also confirmed the deletions reported previously for *CESA3*, *CESA4*, *CESA*8, and *CESA10* (*15*) and *CESA6* and *CESA7* (*18*) by sequencing (figs. S4 and S5). In addition to the chromosome-scale *P. patens* genome assembly (*19*), a near telomere-to-telomere genome sequence is now available (*20*). A similarity search of this sequence revealed no additional CESA sequences (see the Supplementary Materials).

### Moss plants lacking CESAs produce microfibrillar cellulose

**Fig. 2.**
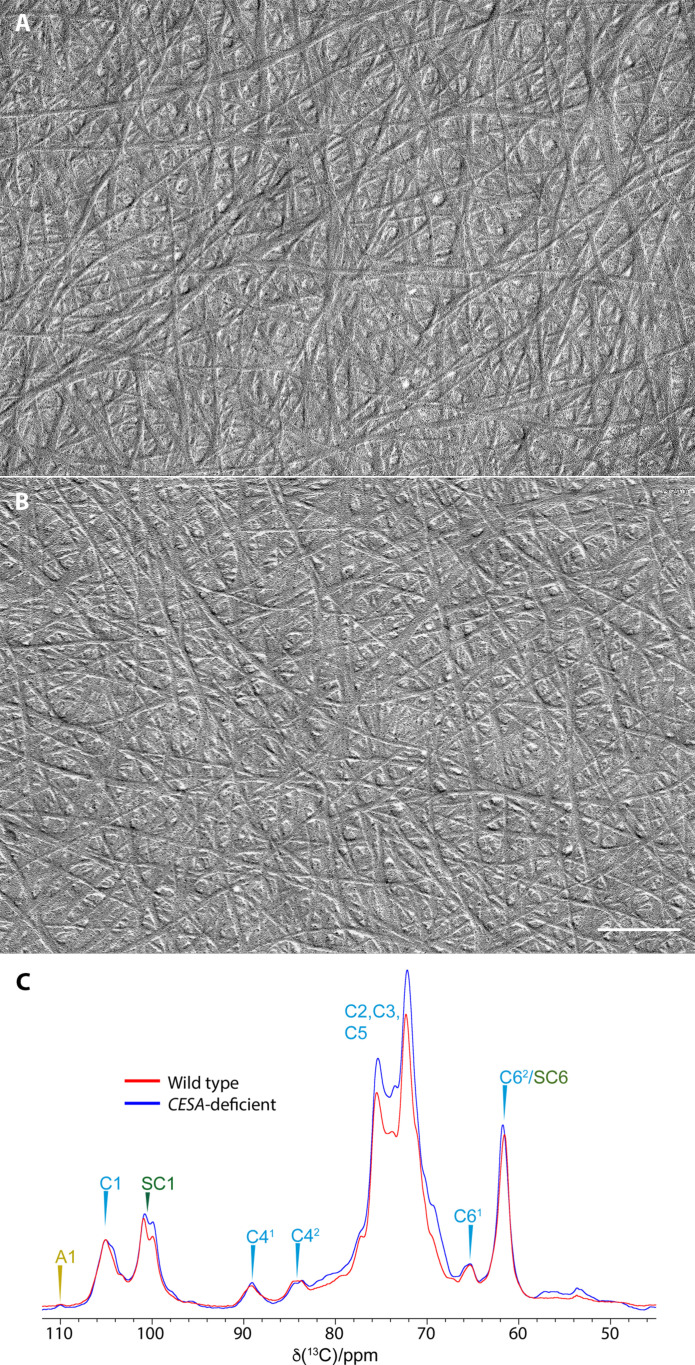
Cellulose microfibrils in wild-type and CESA-deficient *P. patens*. Cell walls were extracted from protonemal filaments of (**A**) wild-type and (**B**) CESA-deficient *P. patens* with 1 N NaOH and acetic-nitric reagent before air-drying and shadowing to reveal microfibrils. (**C**) Neutral carbohydrate region of 1D ^13^C CP-MAS-NMR spectra of CESA-deficient and wild-type *P. patens*. Identifiable ^13^C NMR shifts of cellulose (C), arabinose (A), and starch (SC) are labeled. The spectra were recorded at a ^13^C Larmor frequency of 150.7 MHz and a MAS frequency of 12 kHz. ppm, parts per million.

In addition to demonstrating that cellulose synthesized by CESAs is not required for protonemal tip growth, CESA-deficient *P. patens* lines provide a unique biological tool for investigating the structure of the β-1,4-glucan presumably synthesized by CSLDs. To examine how loss of CESAs affects the fibrillar structure of the cell walls, we extracted protonemal filaments of wild-type and CESA-deficient *P. patens* with 1 N NaOH, followed by acetic-nitric reagent to remove matrix polysaccharides and proteins, and shadowed the residue with platinum carbon. The extracted cell walls were fibrillar (Fig. 2) with no visible differences between wild-type and CESA-deficient genotypes. X-ray diffraction patterns of extracted cell walls from wild-type and CESA-deficient protonemal filaments contained the 110 (15.7°), 200 (22.6°), and 004 (35.19°) peaks characteristic of cellulose (fig. S6, A and B). Similarly, the fluorescent cellulose-binding dye Pontamine fast scarlet 4B (S4B) (*21*) stained the extracted cell walls of both wild-type and CESA-deficient protonemal filaments. Staining intensity was similar in both genotypes and highest in cross walls (fig. S6C).

We used 1D ^13^C cross-polarization–magic angle spinning–nuclear magnetic resonance (CP-MAS-NMR) spectroscopy for structural analysis of untreated cells from CESA-deficient and wild-type *P. patens*. Cellulose and starch dominate the CP-MAS-NMR spectra (Fig. 2C). Peaks labeled C4^1^ and C4^2^ are indicative of cellulose fibrils (*22*). The C4^1^ signals arise from glucosyl residues mostly in crystalline cellulose internal to fibrils and have the C6 hydroxymethyl in the *tg* configuration. The C4^2^ signals arise mostly from glucosyl residues on the surface of cellulose fibrils and have the C6 hydroxymethyl in the *gt/tt* configurations (*22*). The similar strength of both signals suggests the fibrils have similar dimensions to fibrils found in vascular plant primary and secondary cell walls (*23*, *24*).

### Moss plants lacking CESAs have plasma membrane rosettes

The transmembrane (TM) regions of CESA-containing CSCs have been visualized by freeze-fracture transmission electron microscopy (FFTEM) (*25*–*29*). In this technique, cells are frozen rapidly and fractured under vacuum to expose integral membrane proteins, which are typically revealed on the interior surface of the membrane leaflet adjacent to the cytoplasm after the outer leaflet is removed during cell fracture. The fractured specimens are shadowed with platinum/carbon to produce replicas so that the original cell structure becomes interpretable in TEM (*30*). FFTEM has previously shown that the plasma membranes of *P. patens* protonemal filaments contain rosette structures with six particles that often appear triangular (*2*, *31*), similar to the CESA-containing CSCs of vascular plants (*28*).

**Fig. 3.**
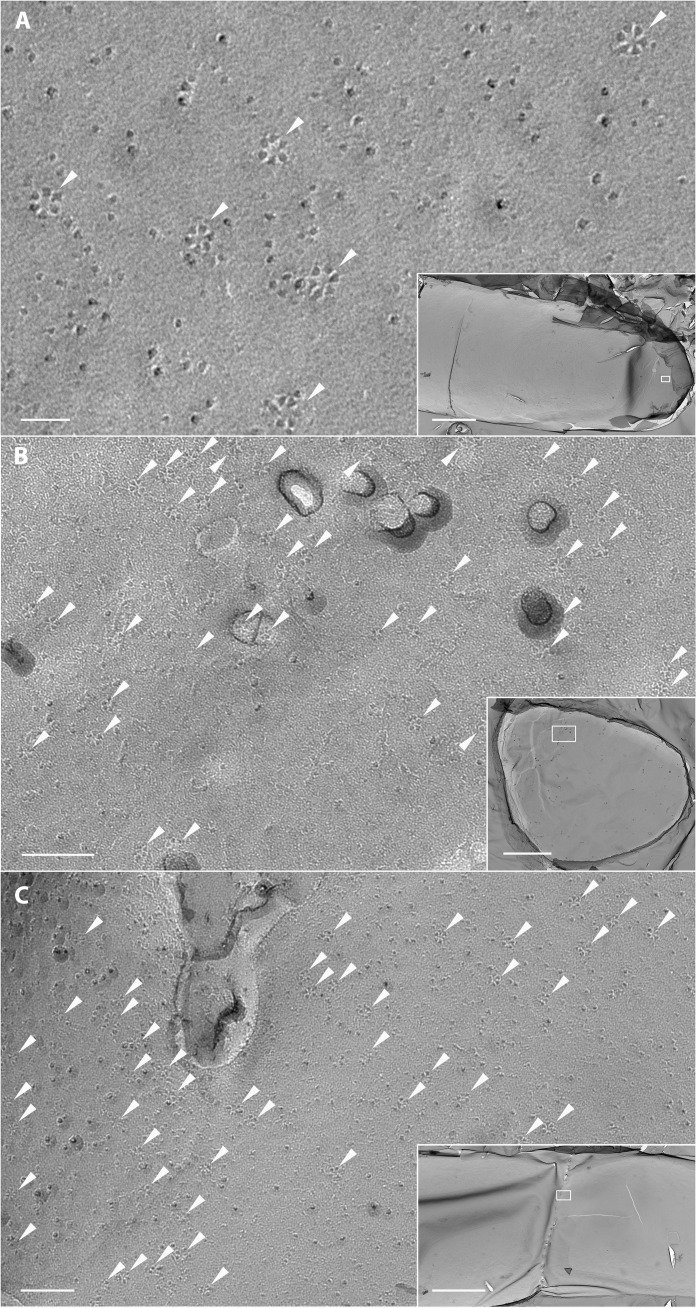
TEM imaging of rosettes in freeze-fracture replicas of CESA-deficient *P. patens*. (**A**) Plasma membrane region from the apex of a protonemal filament (box in inset) with rosettes (arrowheads). Scale bars, 40 nm and (inset) 3 μm. (**B**) Plasma membrane region from a protonemal filament (box in inset) viewed tip down with numerous rosettes (arrowheads). Scale bars, 100 nm and (inset) 2 μm. (**C**) Plasma membrane region of a dividing cell with fusing cell plate (box in inset) with numerous rosettes (arrowheads). Scale bars, 100 nm and (inset) 3 μm.

Here, we show that rosettes are present in CESA-deficient *P. patens* protonemal filaments (Fig. 3A). Because the plane of fracture cannot be readily controlled, we inferred the cellular context of the fractured membranes from cellular landmarks to identify regions with high densities of rosettes. We observed elongated membrane patches representing longitudinal fractures of protonemal plasma membranes including some with rounded ends (Fig. 3A), consistent with fracturing at or near the apex of protonemal tip cells. We also observed circular and oval membrane patches consistent with fractures through the apical plasma membrane of protonemal tip cells oriented perpendicular to the plane of fracture (Fig. 3B). Figure 3C shows a rare fracture that captures the fusion of a cell plate with the parental cell wall. The rosettes observed in our samples were concentrated at the cell tips (Fig. 3B) and adjacent to fusing cell plates (Fig. 3C). These are the same regions where CSLDs have been localized by live-cell imaging in *P. patens* (*10*). Higher magnification views of rosettes in Fig. 3 (B and C) are shown in fig. S7.

### Rosettes from CESA-deficient *P. patens* are morphologically indistinguishable from CESA rosettes

**Fig. 4.**
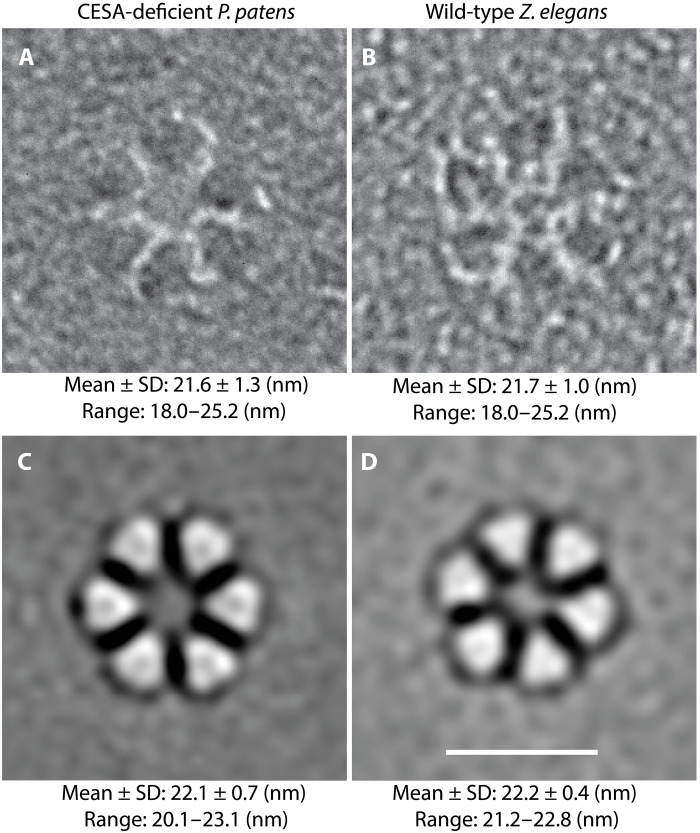
Original images and corresponding image averages of rosettes from two cell types with diameter measurements. (**A**) Original FFTEM images and data from hand measurement of 543 rosettes from nine CESA-deficient *P. patens* protonemal cells (three cells from each of three independent genetic lines) frozen while synthesizing primary cell walls. (**B**) Original TEM image and data from hand measurement of 380 rosettes from five differentiating tracheary elements frozen while synthesizing secondary walls via CESAs. (**C** and **D**) Representative image averages of the rosettes measured in (A) and (B). The contrast of the original images was reversed before reference-free image averaging to accommodate the design of the EMAN2 program. Data for each cell type are from hand measurement of the 42 image averages (six class averages within each of seven refinements). Scale bar, 20 nm.

To test for structural difference between the rosettes from CESA-deficient *P. patens* and CESA-containing CSCs (Fig. 4), we compared *P. patens* mutants with differentiating tracheary elements from *Zinnia elegans* suspension cultures, which synthesize banded secondary cell walls via the activity of abundant CESA-containing rosette CSCs (*32*–*34*). We chose *Z. elegans* suspension cultures for this comparison because the role of CESAs in secondary cell wall deposition in these cultures is well documented (*32*), and freeze-fracture is feasible (*33*, *35*). Although the role of CESAs in *P. patens* leafy gametophore development is also well documented (*15*, *16*), we were unable to produce freeze-fracture replicas of plasma membranes from *P. patens* gametophores because fracture invariably occurred within the cuticle.

We analyzed rosettes in replicas of *Z. elegans* cells that were frozen in the earliest stage of banded secondary cell wall synthesis, when the plasma membrane was still relatively flat, to reduce measurement errors resulting from varied de facto shadowing angles arising from changing topography of the plasma membrane. We measured the external diameters of rosettes in these two cell types manually and used EMAN2 (*36*) to generate 42 reference-free class averages (six class averages within each of seven refinements) in each case. The appearance, mean diameter, and the ranges of diameters of rosettes were similar for CESA-deficient *P. patens* protonemal filaments and cultured *Z. elegans* cells synthesizing secondary walls via CESAs (Fig. 4). Differences in means between cell types were less than the minimum 1.25 nm grain size of FFTEM replicas prepared by these methods (*2*). Image averaging consistently reduced the range and increased the mean diameter by 0.5 nm, probably due to (i) combining smaller rosettes with ones that were slightly larger in class averages and (ii) diminishing the contribution of fewer large rosettes to class averages.

## DISCUSSION

In biology, form follows function. The uniform structure of cellulose microfibrils in land plant cell walls was previously attributed to their synthesis by CESA enzymes arranged in distinctive rosette CSCs. Our results show that non-CESA proteins, most likely CSLDs, also form rosettes and produce microfibrils indistinguishable from those made by CESAs. This finding was possible because, in contrast to *Arabidopsis* (*7*), *P. patens* does not require CESA activity for viability, so mutants expressing CSLDs in the complete absence of CESAs could be obtained. Although CESAs are required to maintain cell integrity in diffuse-growing *P. patens* gametophore buds (Fig. 1), mutant lines in which all eight *CESA* genes are disabled can be propagated as tip-growing protonemal filaments (Fig. 1 and figs. S1 to S4). In contrast, vascular plants such as *Arabidopsis* propagate through diffuse-growing embryos that require CESA activity (*37*, *38*), whereas their tip-growing root hairs and pollen tubes are determinant.

Several lines of evidence support our hypothesis that the rosettes observed in CESA-deficient *P. patens* are formed by CSLDs. Similar to CESAs, CSLDs synthesize β-1,4-glucan (*8*), move in the plasma membrane (*10*), and assemble into particles in vitro that resemble CESA trimers (*8*). In CESA-deficient *P. patens*, rosettes are concentrated at cell tips and adjacent to fusing cell plates (Fig. 3), which is consistent with the distribution of CSLDs previously observed in wild-type *P. patens* using confocal fluorescence microscopy (*10*). Tip-growing protonemal filaments also depend on CSLDs to maintain cell integrity (*10*), as do tip-growing root hairs and pollen tubes of *Arabidopsis* (*39*, *40*). Last, the *P. patens* CESA superfamily includes only two other families (*14*), neither of which is likely to participate in cellulose microfibril formation. These include CSLAs, which synthesize mannan in *P. patens* and vascular plants (*41*), and CSLCs, which, along with CSLAs, function in the Golgi in *Arabidopsis* (*42*). *P. patens* has eight *CSLD* genes that diversified independently from the vascular plant *CSLD* family (*14*). *CSLD2* and *CSLD6* are preferentially expressed in gametophores and are redundantly required for normal cytokinesis. However, all eight CSLD proteins localize to protonemal cell plates and cell tips (*10*). It is unknown whether the *P. patens* CSLDs form homo-oligomeric or hetero-oligomeric complexes.

Our results indicate that the structure of cellulose microfibrils and rosette CSCs have been conserved in parallel throughout the divergence of CESAs and CSLDs, the radiation of land plants, and the specialization of primary and secondary cell walls. On the basis of analysis by TEM, x-ray diffraction, and solid-state NMR, the cellulose microfibrils in CESA-deficient *P. patens* protonemal filaments are structurally indistinguishable from vascular plant microfibrils synthesized by CESAs (Fig. 2 and fig. S4). Similarly, rosettes in CESA-deficient *P. patens* and CESA-containing CSCs in differentiating *Z. elegans* tracheary elements are morphologically indistinguishable based on original FFTEM images and image averages (Fig. 4). Measurements of both conform to rosettes analyzed previously in wild-type *P. patens* protonemal filaments. Original images of 324 wild-type protonemal rosettes had a mean diameter of 21.4 ± 1.3 nm with a range of 17.6 to 25.6 nm, and six EMAN2 image averages had a mean diameter of 22.7 ± 0.5 nm (*2*). In retrospect, we believe that these included both CESA- and CSLD-containing rosettes based on live-cell imaging data for independently tagged CESA and CSLD proteins (*10*).

**Fig. 5.**
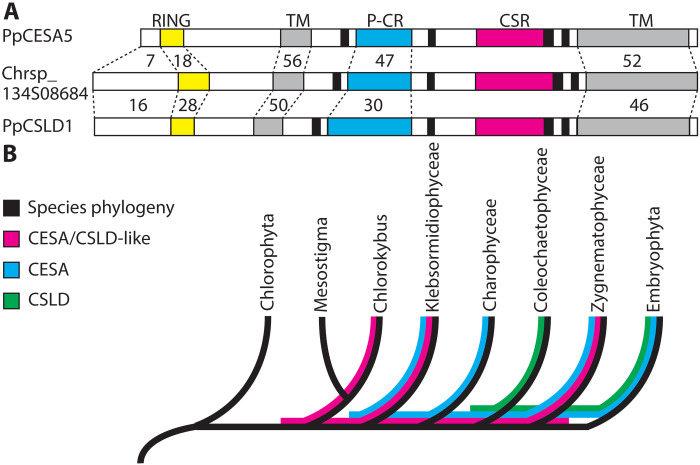
Sequence comparison and lineage sorting of CESAs, CSLDs, and CESA/CSLD-like proteins. (**A**) Graphical comparison between *P. patens* CSLD and CESA protein sequences and a representative CESA/CSLD-like protein from *C. atmophyticus* (Chrsp_134508684). Numbers indicate the percent amino acid identity between PpCESA5 or PpCSLD1 and the *C. atmophyticus* sequence in different regions (dashed lines). CSLDs share greater identity with CESA/CSLD-like sequences in the N-terminal region and RING domain (yellow), but they are more similar to CESAs in the plant-conserved region (P-CR; cyan). Gray = TM regions, black = catalytic “D” and “QxxRW” domains, and magenta = class-specific region (CSR). (**B**) Evolutionary relationships of chlorophyte green algae, five classes of charophyte green algae, and land plants (Embryophyta) depicting incongruence of the CESA, CSLD, and CESA/CSLD-like sequence trees. Evolutionary relationships (topology only) are from (*68*). Sequence distributions are from (*45*–*47*).

FFTEM images reveal the TM region of membrane-associated protein complexes (*30*). The TM regions of CESAs and CSLDs are highly conserved (Fig. 5A and fig. S8), consistent with both enzymes having a glucan translocation channel surrounded by seven TM helices (*1*). Although the TM regions are highly conserved, CESA and CSLD sequences diverge in their cytosolic regions, including the length of the N terminus, the cysteine spacing in the RING domain, and the presence of several insertions in the plant-conserved region (Fig. 5A and fig. S8). The plant-conserved region is a CESA trimerization domain (*1*), and the N terminus, specifically the RING domain, has also been implicated in CESA-CESA interaction (*43*). However, these differences evidently do not affect the ability of CESAs and CSLDs to assemble as rosettes or the particle spacing in the TM domain visualized by FFTEM. The similar spacing of CESA- and presumed CSLD-containing rosette particles (Fig. 4) and their included translocation channels, combined with the structural similarity of the microfibrils they produce (Fig. 2), is consistent with the well-established correlation between CSC organization and cellulose microfibril structure (*27*). We can only speculate whether this apparent uniformity in rosette CSC morphology, despite originating from different gene families, has resulted from purifying selection for microfibril properties that conferred fitness or from genetic constraints that prevented the emergence of new microfibril traits.

On the basis of phylogenetic analysis of angiosperm sequences, CESAs and CSLDs were originally assigned to different families within the CESA superfamily (*44*). Recent analyses incorporating representatives of the major green algal lineages (*45*–*47*) show that CESAs and CSLDs diverged as independently evolving families at least 500 million years ago (Fig. 5). Along with CESA/CSLD-like sequences from charophyte green algae (*45*–*49*), these are the only CESA superfamily members that have the RING domain, plant-conserved region, and class-specific region (Fig. 5A and fig. S8) that form the interfaces between CESAs within rosette CSCs (*1*, *43*, *50*, *51*). Notably, the CESA/CSLD-like sequences have a CESA-like plant-conserved region and a CSLD-like N terminus (Fig. 5A and fig. S8). This, along with phylogenetic occurrence (Fig. 5B), indicates that the CESAs and CSLDs evolved independently from a common ancestral CESA/CSLD-like protein, with CESAs undergoing a reduction of the N terminus and CSLDs acquiring inserts within the plant-conserved region. The gene family trees are discordant with the species tree, with CSLDs absent from Zygnematophyceae but present in the last common ancestor (LCA) shared with Coleochaetophyceae. Similarly, Coloechaetophyceae appears to have lost its *CESA*s after it diverged from the Charophyceae, and the wall-less *Mesostigma viride* appears to have lost its *CESA/CSLD-like* gene after it diverged from its LCA with *Chlorokybus atmophyticus*. This is consistent with the observation that gene family loss was common throughout plant evolution (*52*). Given their similarities to CESAs and CSLDs, it is possible that the CESA/CSLD-like proteins form rosettes. This would place the evolution of the rosette CSC early in the streptophyte lineage (i.e., the clade that includes charophyte green algae and land plants; Fig. 5B).

As discussed recently by Yang *et al.* (*8*), the maintenance of separate CESA and CSLD families in all land plant lineages suggests that each family serves some special function. In *P. patens* and seed plants, CSLDs deposit cellulose in growing cell tips and cell plates (*8*, *10*, *53*), in both cases contributing to synthesis of a wall where none existed (*54*). It was suggested that CSLDs may produce structurally distinct cellulose microfibrils that interact with callose or other cell wall polymers to support tip growth and cell plate development (*8*, *10*, *54*). This now seems less likely given the similarity between CESA- and presumed CSLD-containing rosettes and the cellulose microfibrils they produce. However, CESAs and CSLDs do differ in their plasma membrane movements, interactions with microtubules (*10*), and sensitivity to cellulose biosynthesis inhibitors (*8*, *10*), and they may differ in their tolerance of high Ca^2+^ concentrations (*54*). Tip growth and cytokinesis are both associated with Ca^2+^ gradients (*55*), and CSLDs might be needed for these processes if CESA activity is inhibited by high Ca^2+^ (*54*). In contrast to CESAs (*11*), CSLD movements in the plasma membrane do not track along microtubules, and they are faster and less linear than CESA movements (*10*). This may help maintain cell integrity during rapid isodiametric apical expansion in root hairs, pollen tubes, and protonemal filaments, and for cell plate development, all cases where microfibril deposition need not be oriented. CESAs interact with microtubules through cellulose synthase–interacting protein 1 (CSI1) (*56*). Although the CESA-CSI1 interface has not been identified, evidence suggests that it may reside within the catalytic domain (*56*) and/or the N terminus (*1*). This coincides with sequence divergence between CESAs and CSLDs in the plant-conserved region of the catalytic domain and most of the N terminus (fig. S8). Last, note that the cellulose biosynthesis inhibitor isoxaben inhibits the activity of CESAs, but not CSLDs (*8*, *10*). CSLDs share nearly all of the amino acids for which point mutations confer isoxaben resistance in CESAs (*10*), adding to the questions that have been raised about cellulose biosynthesis inhibitor mechanism of action (*57*). Future examination of CESA and CSLD interaction with microtubules and sensitivity to Ca^2+^ and isoxaben promises to shed light on the evolution of regulatory differences and their consequences for cellulose microfibril biosynthesis by distinct protein families at different stages of plant cell development.

## MATERIALS AND METHODS

### Culture of *P. patens*

For routine subculturing and to generate tissue for transformation, rapid freezing, and cell wall isolation, we incubated cultures at 24°C with constant illumination at 50 to 80 μmol m^−2^ s^−1^ in a plant tissue culture incubator (model CU36L5, Percival Scientific Chambers, Perry, IA, USA). We subcultured filaments weekly on basal medium supplemented with ammonium tartrate [BCDAT: 1.0 mM MgSO_4_, 1.9 mM KH_2_PO_4_, 10 mM KNO_3_, 45 μM FeSO_4_, 5.0 mM diammonium tartrate, 1 mM CaCl_2_, 220 nM CuSO_4_, 190 nM ZnSO_4_, 10 μM H_3_BO_3_, 2.0 μM MnCl_2_, 230 nM CoCl_2_, 170 nM KI, and 100 nM Na_2_MO_4_ solidified with 0.7% (w/v) agar] and overlain with cellophane (*58*). For solid-state NMR, we cultured filaments on solid BCDAT medium at 21°C under 16-hour/8-hour day/night cycle and subcultured them on BCDAT medium supplemented with 1% ^13^C glucose to obtain the ^13^C-labeled tissues.

### *Z. elegans* seedling growth

We stored seeds of *Z. elegans* L. var. Envy (A5896 N; Grimes Seeds, Concord, OH, USA) in the refrigerator under desiccation until planting. We planted seeds by dispersing them (3.5 g) uniformly on a tray of moist potting mix (Sunshine Mix #8/Fafard-2 with RESiLIENCE, Sun Gro Horticulture, Agawam, MA, USA), covering them lightly, and germinated them in a growth chamber (Model AR36L, Percival Scientific Chambers) with a 16-hour/8-hour, 28°/24°C day/night cycle and 50% relative humidity. Light intensity was 170 μmol m^−2^ s^−1^ at tray height generated by fluorescent and incandescent lamps. We placed seedling trays with drainage holes inside trays without holes and watered them 3 days a week by flooding the outer tray for about 30 min and then pouring out excess water.

### Generation and verification of CESA-deficient *P. patens* lines

We constructed the CRISPR-Cas9 CESA5 knockout (KO) vector as described previously (*15*, *59*). We designed protospacers (table S1) targeting two sites within *CESA5* (Phytozome identifier: Pp3c2_13330V3.1) using CRISPOR at http://crispor.tefor.net/ (*60*) and cloned the protospacers into entry vectors pENTR-Ppu6p-sgRNA-L1R5 and pENTR-Ppu6p-sgRNA-L5L2 (Addgene; www.addgene.org/) for tandem insertion into the destination vector. After annealing protospacers as described previously (*59*), we ligated them into pENTR-PpU6p-sgRNA entry vectors using Golden Gate assembly (New England Biolabs, Ipswich, MA, USA) in 10-μl reactions containing 19 fmol of entry vector and 35 fmol of annealed protospacer incubated at 37°C for 1 hour and 60°C for 5 min (*10*). We recombined the resulting entry vectors with destination vector pZeo-Cas9-gate (Addgene), which confers zeocin resistance, using Invitrogen LR Clonase II Plus according to the manufacturer’s instructions (Thermo Fisher Scientific, Waltham, MA, USA). We sequence-verified all plasmids. The construction of the CESA1KO vector was described previously (*15*).

We edited a previously described *cesa6/7/3/8/10/4* KO-41 line (*15*) with CRISPR-Cas9 to disable *CESA5* and *CESA1*. As described previously (*58*), we transformed protoplasts generated from filaments cultured on solid BCDAT medium with CRISPR-Cas9 KO vectors and selected colonies for genotyping after one round of selection on Zeocin (50 μg ml^−1^) or hygromycin (15 μg ml^−1^) (*59*). We isolated genomic DNA as described previously (*58*) and amplified it with primers (table S1) flanking the target sites and potential off-target sites predicted by CRISPOR (*60*). We analyzed the PCR products by gel electrophoresis to identify large deletions and sequenced them to confirm editing at target sites and the absence of editing at off-target sites.

For additional verification of the KO genotype, we used primers designed to amplify deletions in *CESA1*, *CESA3*, *CESA4*, *CESA5*, *CESA8*, and *CESA10* (table S1) to amplify genomic DNA extracted from wild-type and final CESA-deficient lines. The background line used for the first round of CRISPR mutagenesis (*15*) was *cesa6/7*KO-1 produced by homologous recombination (*18*), and we verified the deletion of these two genes by PCR in the final CESA-deficient line (fig. S3). We also sequenced fragments amplified with primers flanking the deletions in *CESA3*, *CESA4*, *CESA8*, and *CESA10* (table S1 and fig. S4) and the entire *CESA6/CESA7* tandem pair (fig. S5) to confirm that the final CESA-deficient lines retained the deletions described previously for the *cesa6/7/3/8/10/4* KO-41 line (*15*).

Last, we downloaded gene models from the near telomere-to-telomere sequence of *P. patens* (*20*) and searched them by blastp in Geneious Prime v. 2019.2.3 using PpCESA5 (Phytozome peptide: Pp3c2_13330V3.1) as a query and the BLOSUM62 matrix with a max *E* value = 10. All hits were matched with their corresponding Phytozome gene model by blastp search.

### Time-lapse imaging of developing gametophore buds

To image developing gametophores of CESA-deficient mutants, we pipetted ground protonemal tissue into the central part of microfluidic imaging chambers (*61*) and submerged them in half-strength Hoagland’s medium [2 mM KNO_3_, 1.0 mM KH_2_PO_4_, 0.50 mM Ca(NO_3_)_2_, 45 μM Fe citrate, 150 μM MgSO_4_, 5.0 μM H_3_BO_3_, 110 nM CuSO_4_, 1.0 μM MnCl_2_, 115 nM CoCl_2_, 95 nM ZnSO_4_, 85 nM KI, and 51 nM Na_2_MoO_4_]. We incubated the chambers under constant illumination at 85 μmol m^−2^ s^−1^ for 2 weeks before imaging. We acquired time-lapse images on a Nikon TIE body equipped with a Plan Apo λ 20× objective and a Nikon DS-Fi2-L3 camera (Nikon Instruments, Melville, NY, USA).

### Extraction of cellulose microfibrils

For extraction of cellulose microfibrils, we harvested 7-day-old filaments from solid medium, ground them in a mortar under liquid nitrogen, extracted them with 1 N NaOH at 100°C for 1 hour, and washed them with filtered water to neutrality. We extracted the NaOH-insoluble fraction with acetic-nitric reagent (*62*) at 100°C for 1 hour and then collected insoluble material by centrifugation. We re-extracted the pellet in fresh acetic-nitric reagent for 30 min at 100°C, washed with filtered water to neutrality, and then stored the material frozen or air-dried for x-ray analysis.

### Transmission electron microscope imaging of metal-shadowed cell walls

For metal shadowing, we suspended the 1 N NaOH and acetic-nitric reagent-extracted material in distilled water and pipetted it onto freshly cleaved mica and allowed it to air-dry in a dust-free environment. We clamped these samples onto a single-replica freeze-fracture sled and inserted them into a Cressington model 308 R freeze-fracture apparatus (Cressington Scientific Instruments, Watford, UK) at room temperature. After high-vacuum conditions were established, we shadowed the sample with platinum/carbon in the same manner as freeze-fractured samples (see below). After removing the samples from the freeze-fracture machine, we scored the mica with a pin and immersed the samples in chromic-sulfuric acid. While some replicated regions detached quickly, most did not. After several hours, we were able to detach replica fragments using an acid stream expelled from a drawn-out glass Pasteur pipet. We transferred the replicas through distilled water washes using a platinum loop, picked them up on Formvar-coated copper grids, and imaged them in the same manner as freeze-fracture replicas (see below).

### X-ray diffraction

For x-ray diffraction, we formed thin circular membranes by collecting NaOH and acetic-nitric reagent extracted material by suction onto nylon filters (5-μm-pore size), peeling the insoluble material from the filter and then drying them in a dust-free environment. We used a Rigaku SmartLab x-ray diffractometer operating at 40 kV, 44 mA (Cu Kα radiation) to generate diffractograms from these paper-like samples.

### Staining with S4B

We stained protonemal filaments extracted with acetic-nitric reagent without grinding with 0.01% S4B (*21*) in tris-buffered saline and examined them with an epifluorescence microscope (Olympus BH-2 with green filter set with 405-nm excitation and 455-nm dichroic mirror and barrier filter) and a confocal scanning microscope (Olympus Fluoview FV1000 confocal microscope with UIS2 40× numerical aperture 1.3 oil immersion objective and 559-nm diode laser). We captured epifluorescence images using a Q-Color5 camera (Olympus America, Central Valley, PA, USA).

### Solid-state NMR analysis

We labeled tissue by subculturing it three times (14 days each) on BCDAT solid medium containing 55.6 mM ^13^C glucose. After snap freezing on dry ice and thawing, we packed the tissue into the rotor and wicked away excess water. We performed solid-state MAS NMR on a Bruker (Karlsruhe, Germany) Avance Neo solid-state NMR spectrometer, operating at ^1^H and ^13^C Larmor frequencies of 600 and 150.7 MHz using a 3.2-mm double-resonance EFree MAS probe. We conducted all experiments at room temperature at a MAS frequency of 12.5 kHz. We determined the ^13^C chemical shift using the carbonyl peak of alanine at 177.8 parts per million as an external reference with respect to tetramethylsilane. The ^1^H 90° pulse length was 3.0 μs, and we used ^1^H-^13^C CP with ramped (70 to 100%) ^1^H radio frequency amplitude, a 1-ms contact time, and SPINAL-64 decoupling (*63*) with a 2-s recycle delay to acquire the spectrum.

### Preparation of xylogenic *Z. elegans* suspension cultures

Similar to established methods (*64*), we released mesophyll cells from first leaves (about 1 cm long) of 8-day-old *Z. elegans* var. Envy seedlings after sterilization in calcium hypochlorite. We concentrated the cells by gentle centrifugation, washed them in medium, inoculated flasks at the required density (12-ml total volume in 50-ml Erlenmeyer flasks), and then cultured them for 2 days at 27°C with 93 rpm rotary shaking in the dark. We observed early banded secondary cell wall thickenings 48 to 49 hours after culturing using an Olympus BH-2 epifluorescence microscope (violet filter set with 405-nm excitation and 455-nm dichroic mirror and barrier filter) after addition of a cellulose-binding fluorophore [Tinopal LPW, Ciba Geigy, Summit, NJ, USA; 0.0005% (w/v) final concentration] to a small drop of the cells in medium. We used an additional barrier filter (Zeiss KP560) in the emission light path to block chlorophyll autofluorescence. To increase the frequency of relatively flat bands of rosettes in the freeze-fracture replicas, we collected cells for freezing when the fluorescence of patterned secondary cell wall thickenings was dimly visible.

### Freeze-fracture TEM

We prepared CESA-deficient *P. patens* protonemal filaments for FFTEM as described previously (*2*) with some modifications. We cultured filaments for 7 days on solid BCDAT medium, homogenized them in water using a hand-held tissue homogenizer with a disposable hard tissue probe (Omni International, Kennesaw, GA, USA), and cultured them at low density (approximately 10 mg wet weight of inoculum per plate) for 4 days on the same medium. Colonies were collected with a micro-spatula (Electron Microscopy Sciences, Hatfield, PA, USA) and mounted in 1 μl of bread yeast hydrated in water. We concentrated *Zinnia* cells at an early stage of patterned secondary wall synthesis by gently suctioning them onto a nylon filter (5-μm-pore size), which we placed on medium-saturated filter paper for 1 hour recovery before collecting the concentrated cells for freezing with a micro-spatula (*33*). We froze samples mounted between two thin copper sample holders by plunging them into ultracold propane (EMS-002, Electron Microscopy Sciences) (*2*) and stored specimens in liquid nitrogen until use.

We prepared and cleaned replicas as described previously (*2*). Briefly, we loaded copper planchets into a double replica holder under liquid nitrogen and transferred them to the liquid nitrogen cooled stage of a freeze-fracture machine (model 308R, Cressington Scientific) under vacuum (<1 × 10^−7^ mbar). We warmed the stage to −120°C for 20 min to evaporate propane and cooled it to −150°C for fracturing. We rotary-shadowed the fractured specimens at 60° with 1.2 to 1.6 nm of Pt/C and applied 13 to 15 nm of carbon at 85° with continuous sample rotation. We cleaned replicas with chromic-sulfuric acid, rinsed them in water, and mounted them on Formvar-coated copper grids. We collected digital images of the acid-cleaned replicas with a high-definition complementary metal-oxide semiconductor camera (NanoSprint43 43mp, AMT Imaging, Woburn, MA, USA) at ×80,000 magnification in a transmission electron microscope (Hitachi HT7800 operated at 80 kV, Hitachi High-Tech, Ibaraki, Japan). We used eucentric focus, which generated good correspondence between nominal and actual magnification as verified by measurement of lattice spacings in negatively stained catalase crystals (40800, Ladd Research Industries, Williston, VT, USA).

### Morphometric analysis of rosettes

We analyzed rosettes from images of protoplasmic fracture (PF) faces of the inner surface of the plasma membrane bilayer (*65*), which is revealed when the outer leaflet of the plasma membrane is removed by the fracture process. The PF lacks “hairy” filamentous structures (possibly cellulose fibrils and/or other polymers) that are visible on the exoplasmic fracture face of the plasma membrane adjacent to the cell wall (*66*). As fracture occurs to reveal the PF, the transmembrane helix (TMH) regions (lobes) of rosettes remain attached to the cytosolic region of the complex while pulling out of the outer leaflet so that the entire membrane-spanning regions of the TMHs can be viewed top-down in the replica.

First, a trained investigator examined unmodified digital TEM images (recorded at ×80,000) on a 4K monitor and identified potential rosettes that met the following criteria: (i) groups of five to six particles arranged in a hexagon and (ii) within a flat, uniformly shadowed, and well-focused area of the image. Next, a group of three to five experts working together excluded examples from further analysis when they did not all agree that a particle cluster was likely to be a rosette. Although rosettes are distinctive within plant plasma membranes, this vetting process was carried out because other types of intramembrane particles can form clusters.

We measured each final set of rosettes by hand and by reference-free class averaging with EMAN2 (https://blake.bcm.edu/emanwiki/EMAN2) (*36*). Using the polygon selection tool in Fiji (https://fiji.sc/) (*67*), we enclosed each rosette in a hexagon by anchoring the outer edge of each lobe without omitting parts of any lobe and calculated the estimated long diameter (*d*) from the included area (*A*), assuming the geometry of a regular hexagon: *d* = 1.732 × [SQRT (*A*/2.5982)]. Values for *d* calculated in this way are slightly lower compared to values determined from circular selections, which often include more free space around the lobes (*2*).

### Reference-free class averaging

We used EMAN2 version 2.91 (https://blake.bcm.edu/emanwiki/EMAN2) (*36*) to create averaged images of rosettes from micrographs with image contrast inverted upon import and a scale factor of 1.598 apix. We selected particles manually using e2boxer with a box size of 300 pixels. After performing contrast transfer function (CTF) correction in EMAN2 and specifying a particle set, we performed reference-free class averaging using e2refine2d.py, typically using six classes (number of classes = 6).

## Supplementary Materials

### The PDF file includes:

Supplementary Text Figs. S1 to S8 Table S1 Legends for movies S1 and S2 References
