# Supplementary material for: An alternate route for cellulose microfibril biosynthesis in plants
Source: Sci Adv. 2024 Dec 13;10(50):eadr5188. doi: 10.1126/sciadv.adr5188 (PMC11641006; doi:10.1126/sciadv.adr5188)
Supplement: Supplementary file 1 — Supplementary Text Figs. S1 to S8 Table S1 Legends for movies S1 and S2 References [file sciadv.adr5188_sm.pdf]

Science Advances
AAAS

Supplementary Materials for

An alternate route for cellulose microfibril biosynthesis in plants

Eric M. Roberts *et al.*

Corresponding author: Alison W. Roberts, aroberts@uri.edu

Sci. Adv. **10**, eadr5188 (2024)
DOI: 10.1126/sciadv.adr5188

The PDF file includes:

Supplementary Text
Figs. S1 to S8
Table S1
Legends for movies S1 and S2
References

Other Supplementary Material for this manuscript includes the following:

Movies S1 and S2

Supplementary Text:

CESA-deficient *P. patens* lines

We tested for CRISPR-mediated induction of a deletion in *CESA5* (Phytozome ID: Pp3c2_13330V3.1) in the *cesa6/7/3/8/10/*4KO-41 background (*15*) by PCR and sequencing to confirm three independent septuple *cesa6/7/3/8/10/4/5*KO lines (Fig. S1). Amplification and sequencing of five off-target sites predicted by CRISPOR (*60*) identified no edits. Inactivation of *CESA5* abolished gametophore development but had little effect on protonemal growth (Fig. 1). We tested for CRISPR-mediated induction of a deletion of *CESA1* (Pp3c9_11990V3.1) in the *cesa6/7/3/8/10/4/5*KO-2 background by PCR and sequencing to confirm three independent octuple *cesa6/7/3/8/10/4/5/1*KO lines (Fig. S2). We observed no obvious additional effect on phenotype (Fig. 1). There were no predicted off-target sites. For the sextuple KO background line, CRISPR-mediated deletions and resulting frameshifts were verified by amplifying across the deletions and sequencing the PCR products (*15*). As a further verification of complete CESA knockout, we designed primers to amplify within the CRISPR-mediated deletions. These primer pairs amplified products for all CRISPR-mutated CESAs in *P. patens* wild type, but not the *cesa6/7/3/8/10/4/5/1*KO CESA-deficient lines (Fig. S3). We amplified with control primers targeting *CSLD3* (Table S1) to verify DNA quality for both lines. The background line used for the first round of CRISPR mutagenesis (*15*) was *cesa6/7*KO-1 produced by homologous recombination (*18*), and deletion of these two genes was also verified by PCR (Fig. S1) in the CESA-deficient lines. Finally, we amplified and sequenced the *CESA3* (Pp3c8_7420V3.1), *CESA4* (Pp3c9_2550V3.1), *CESA6* (Pp3c15_7120V3.1), *CESA7* (Pp3c15_7150V3.1), *CESA8* (Pp3c3_34520V3.1), and *CESA10* (Pp3c9_2670V3.1) loci to verify the deletions reported previously for *cesa6/7*KO-1 (*18*) and *cesa6/7/3/8/10/*4KO-41 (*15*) in the CESA-deficient lines (Figs. S4 and S5).

Other CESA sequences in the *P. patens* genome include *CESA2* (Pp3c1_22600V3.1), which contains frameshift mutations as detected in the genome sequence and verified by sequencing of an independent genomic clone (*69*), and *CESA9* (Pp3c10_10270V3.1), an expressed, apparently non-coding gene containing a small CESA fragment. A blastp search of the gene models from the near telomere-to-telomer sequence of *P. patens* using PpCESA5 as a query returned 35 hits with E-values <10. All 25 corresponding gene models matched gene models from the *Physcomitrium patens* v. 3.3 genome in Phytozome (https://phytozome-next.jgi.doe.gov/) and included the eight *CESA*s that we targeted for knockout as described above, the known *CESA2* pseudogene (Pp3c1_22600V3.1) (*14*), two known *CESA* fragments (Pp3c10_10270V3.1, Pp3c16_15210V3.1) (*45*), the eight known *CSLD* genes (Pp3c2_1280V3.1, Pp3c25_12650V3.1, Pp3c1_41250V3.1, Pp3c1_41400V3.1, Pp3c14_26100V3.1, Pp3c6_4060V3.1, Pp3c2_1330V3.1, Pp3c17_22380V3.1) (*10*), and six additional genes that lacked a glycosyltransferase 2-like domain (IPR001173) and were annotated with other functions (Pp3c12_24670V3.1, Pp3c11_23510V3.1, Pp3c23_19630V3.1, Pp3c3_12270V3.1, Pp3c1_3280V3.1, Pp3c9__3670V3.1). No additional CESA gene models were identified.

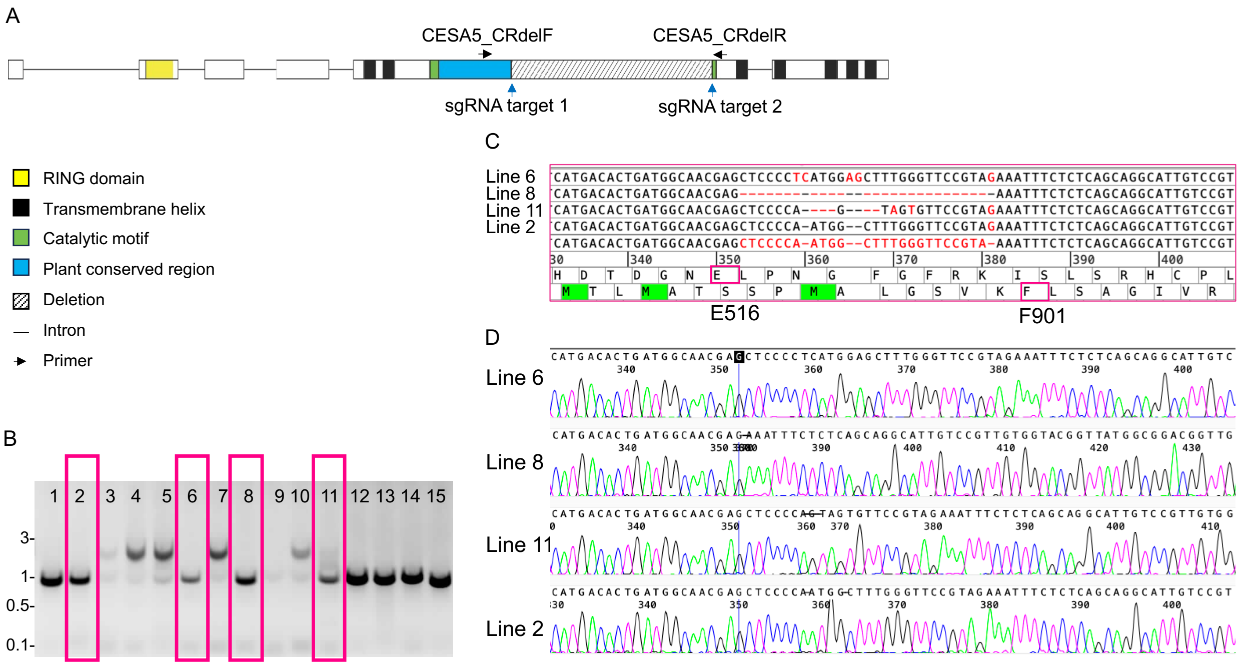

Fig. S1. PCR and sequencing-based genotyping of *CESA5* KO in *cesa6/7/3/8/10/4*KO-41. (**A**) Schematic showing PCR genotyping strategy with primers (black arrows) designed to amplify across two sgRNA target sites (blue arrows). (**B**) PCR products from amplification of genomic DNA extracted from lines selected for transient antibiotic resistance following transformation of *cesa6/7/3/8/10/4*KO-41 (*15*) with a vector targeting *CESA5*. For lines 1, 2, 6, 8, and 11-15, primer pair CESA5_CRdel-F/R (Table S1) amplified a small product consistent with CRISPR-induced deletions in *CESA5*. Deletion of sequence including 60% of the CESA5 catalytic domain (Y345-D719) and introduction of a frameshift were verified by sequencing for lines 2, 6, 8 and 11.

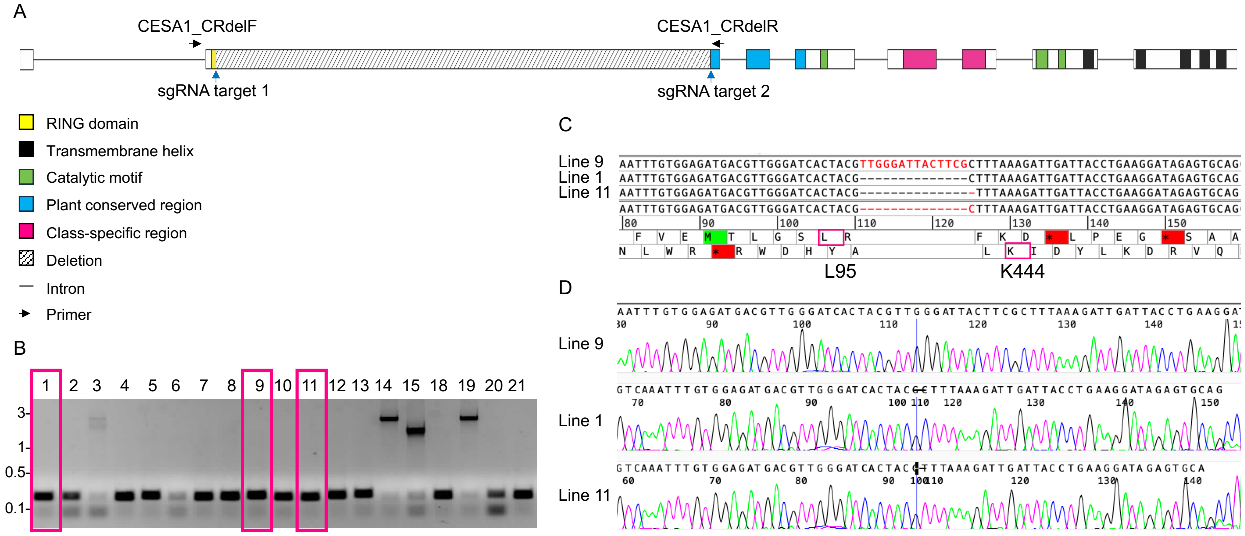

Fig. S2. PCR and sequencing-based genotyping of *CESA1* KO in *cesa6/7/3/8/10/4/5*KO-2. (**A**) Schematic showing PCR genotyping strategy with primers (black arrows) designed to amplify across two sgRNA target sites (blue arrows). (**B**) PCR products from amplification of genomic DNA extracted from lines selected for transient antibiotic resistance following transformation of *cesa6/7/3/8/10/4/5*KO-2 with a vector targeting *CESA1*. For lines 1, 2, 4, 5, 7-13, 18, 20 and 21, primer pair CESA1_CRdel-F/R (Table S1) amplified a small product consistent with CRISPR-induced deletions in *CESA1*. Deletion of sequence including the first half of the CESA1 catalytic domain (L95-K444) and introduction of a frameshift were verified by sequencing for lines 1, 9 and 11.

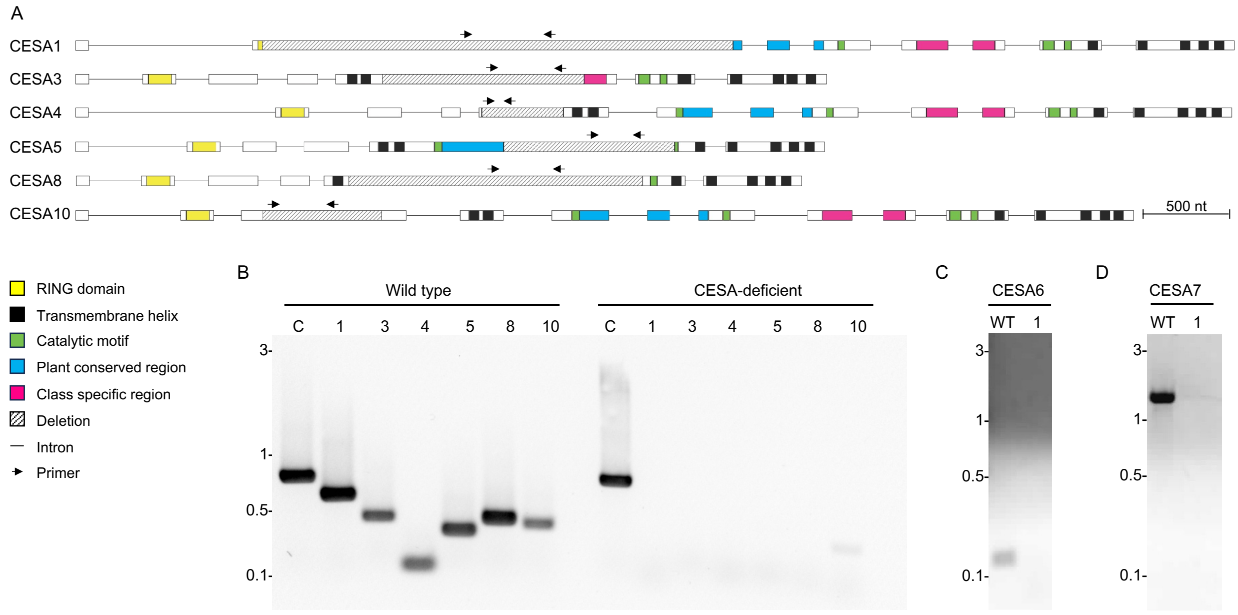

Fig. S3. PCR-based genotyping of the *CESA1, CESA3, CESA4, CESA5, CESA8,* and *CESA10* loci in final *CESA*-deficient *P. patens* lines. (A) Schematic showing the genomic sequences with deletions reported previously (*15*) for *CESA3, CESA4, CESA8,* and *CESA10* and reported here for *CESA1* and *CESA5* with locations of primers used to verify deletions (black arrows). **(B)** PCR products from amplification of genomic DNA extracted from wild type and *cesa6/7/3/8/10/4/5/1*KO-1 with primers targeting sequences in *CESA1*, *CESA3, CESA4, CESA5, CESA8,* and *CESA10* (Table S1). Five primer pairs amplified the expected product from wild type DNA and failed to amplify *cesa6/7/3/8/10/4/5/1*KO-1 DNA. The primer pair targeting *CESA10* amplified the expected product (423 bp) in wild type and weakly amplified a smaller product in *cesa6/7/3/8/10/4/5/1*KO-1, consistent with the presence of similar primer binding sites in *CESA4* (upstream of the *CESA4* deletion) with a predicted amplicon size of 401 bp. DNA quality for *cesa6/7/3/8/10/4/5/1*KO-1 was confirmed by amplification with control primers CSLD3_CRdel-F/ CSLD3_CRdel-R (Table S1). **(C)** PCR products from amplification of genomic DNA extracted from wild type and *cesa6/7/3/8/10/4/5/1*KO-1 with primers targeting *CESA6*. **(D)** PCR products from amplification of genomic DNA extracted from wild type and *cesa6/7/3/8/10/4/5/1*KO-1 with primers targeting *CESA7*. Both *CESA6* and *CESA7* were deleted in their entirety as reported previously (*18*) and shown in Fig. S5.

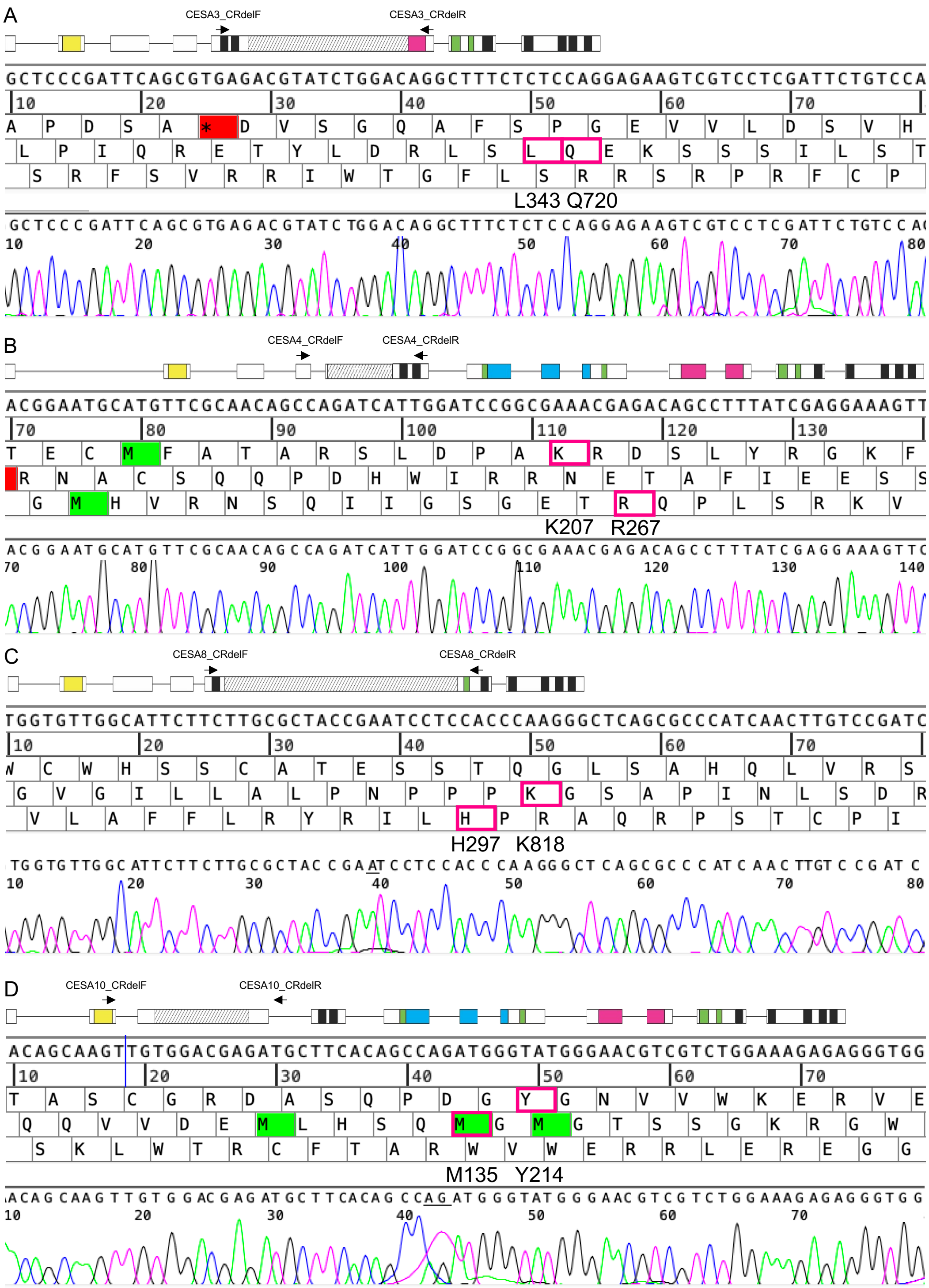

Fig. S4. PCR and sequencing-based genotype confirmation of deletions in the *CESA3, CESA4, CESA8,* and *CESA10* loci in CESA-deficient lines. Schematics showing primers flanking the deletion (black arrows) are shown above sequencing results verifying the deletions reported previously (*15*) for **(A)** *CESA3*, **(B)** *CESA4*, **(C)** *CESA8* and **(D)** *CESA10*.

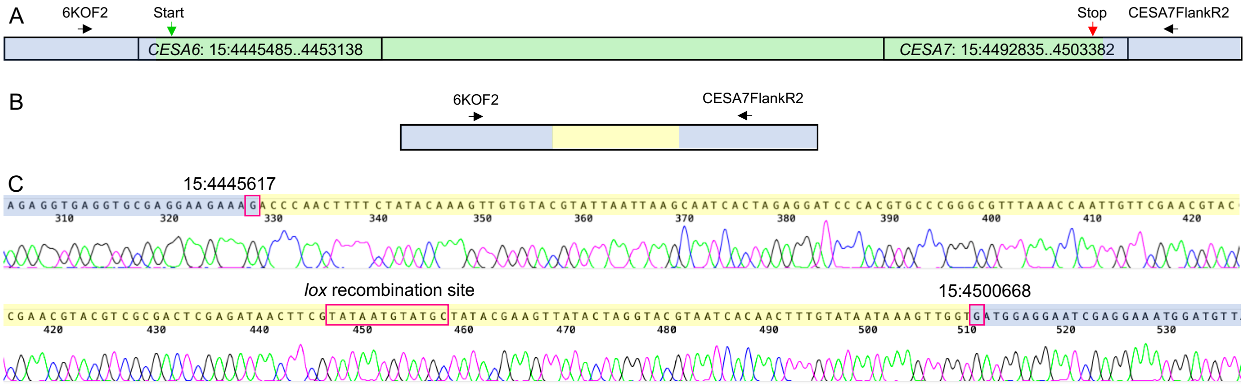

Fig. S5. PCR and sequencing-based genotype confirmation of deletion of the *CESA6* and *CESA7* loci in CESA-deficient lines. (A) Schematic of the CESA6/CESA7 tandem repeat showing genomic coordinates of *CESA6* and *CESA7* from the *P. patens* genome (v6.1, https://phytozome-next.jgi.doe.gov/), primers flanking the deletion (black arrows), *CESA6* start codon, and *CESA7* stop codon. The deleted region is shaded in green and flanking regions are shaded in gray. **(B)** Schematic of the same locus following replacement of *CESA6* and *CESA7* with a selection cassette by homologous recombination (HR) and subsequent removal of the selection cassette by *cre-lox* recombination. **(C)** Sequencing results labeled with the last remaining nucleotides upstream and downstream of the deletion reported previously (*18*). The flanking regions are shaded in gray and the remains of the HR vector, including the lox recombination site, are shaded in yellow.

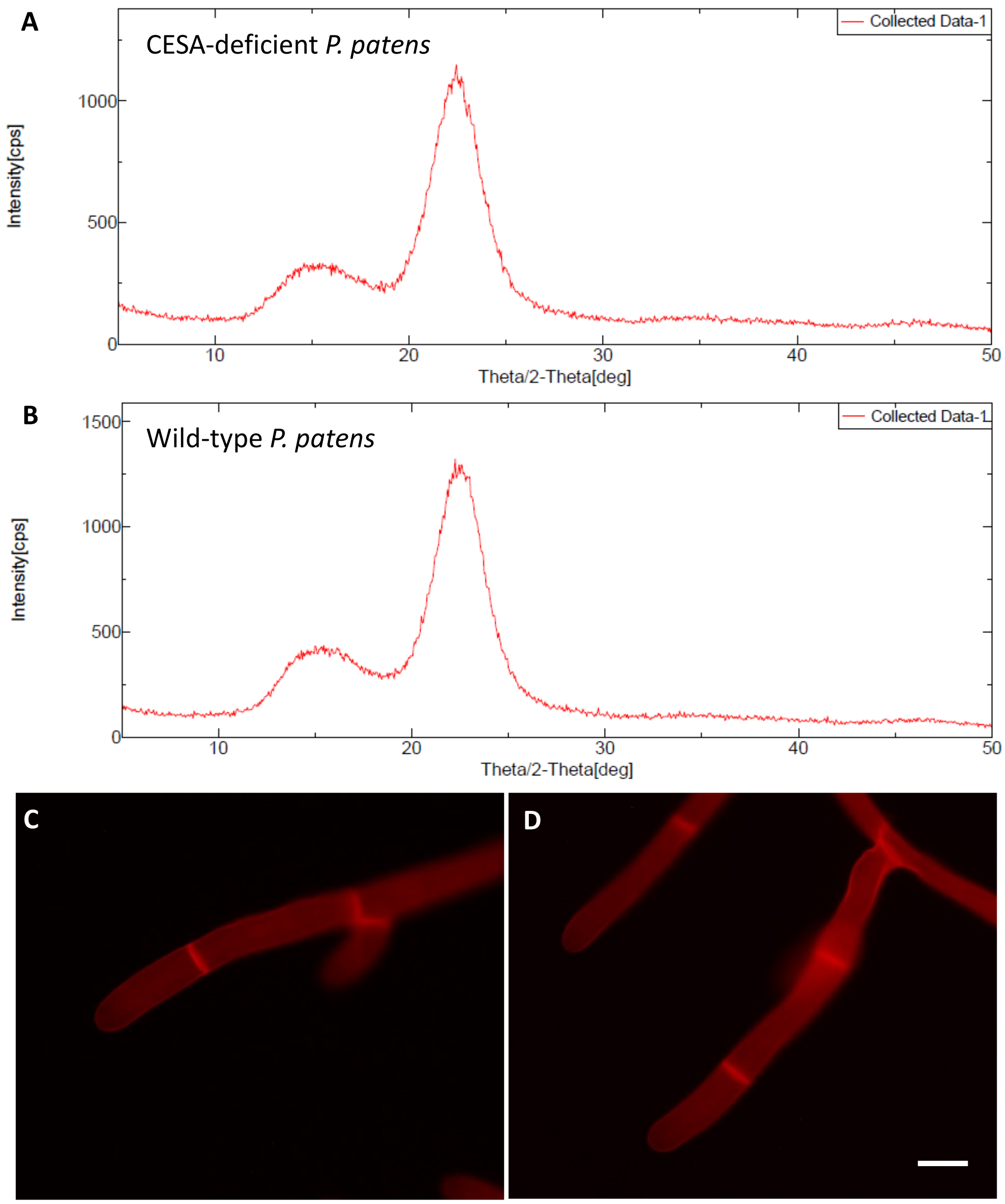

Fig. S6. X-ray diffractograms and Pontamine Fast Scarlet 4B (S4B) staining of cell walls isolated from (A, C) CESA-deficient and (B, C) wild-type *P. patens* protonemal filaments. Tissue was extracted sequentially with 1 N NaOH and acetic-nitric reagent each at 100°C. **(A, B)** The 110 (15.7°), 200 (22.6°) and 004 (35.19°) peaks are characteristic of cellulose. **(C, D)** Staining intensity of extracted cell walls with S4B, a fluorescent dye with high affinity for cellulose (*21*), is similar for wild type and CESA-deficient protonemata and highest in cross walls. Specimens were photographed with epifluorescence optics under identical conditions. We did not detect fibrillar staining when these specimens were examined with confocal laser scanning microscopy. Scale bar = 25 µm.

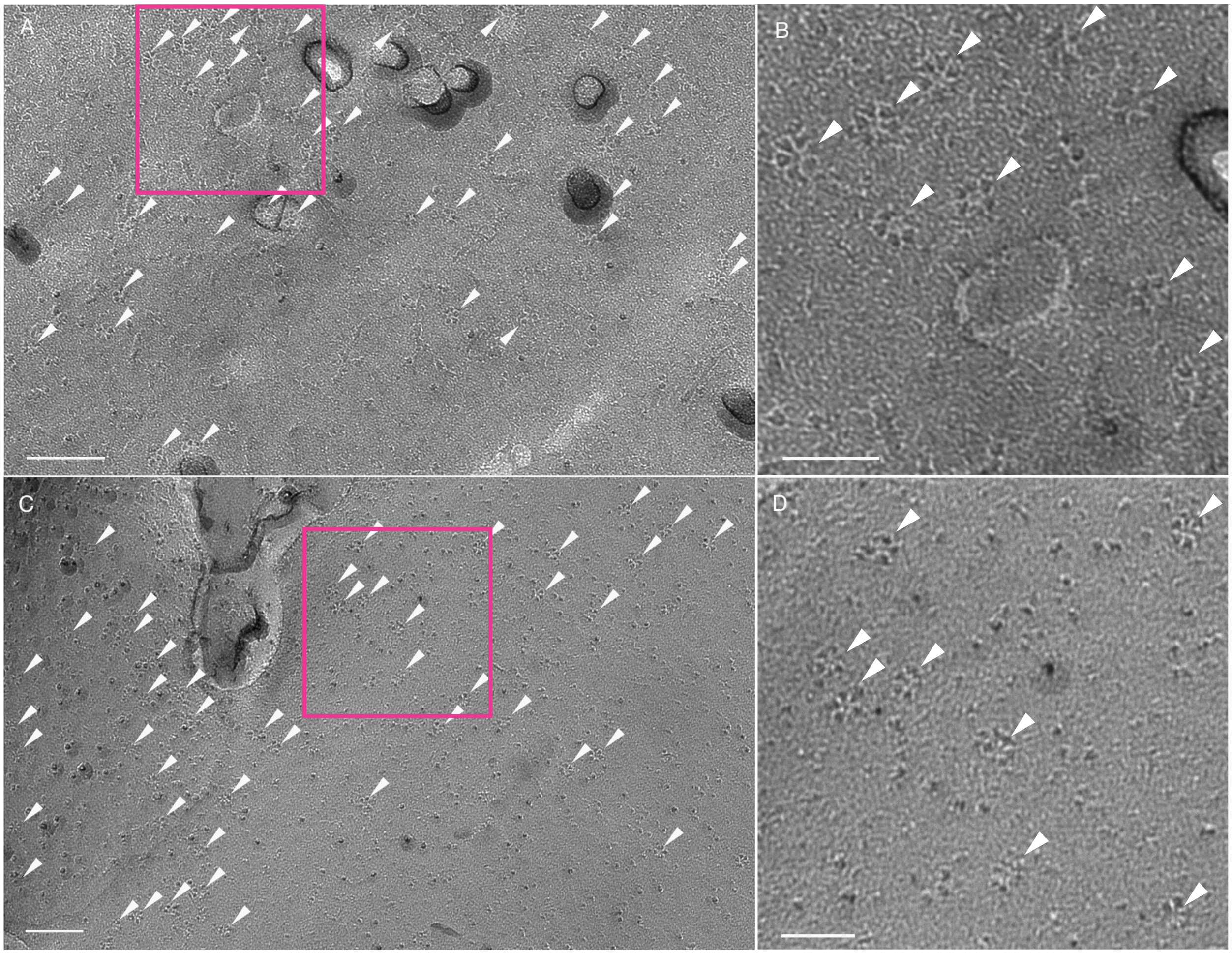

Fig. S7. Higher magnification views of rosettes marked in Figs. 3B and 3C. (A) Image from Fig. 3B with rosettes (arrowheads) and magenta box marking the area of interest. Scale bar = 100 nm. **(B)** Higher magnification view of rosettes within the magenta box in **A**. Scale bar = 50 nm. **(C)** Image from Fig. 3C with rosettes (arrowheads) and magenta box marking the area of interest. Scale bar = 100 nm **(B)** Higher magnification view of rosettes within the magenta box in **C**. Scale bar = 50 nm.

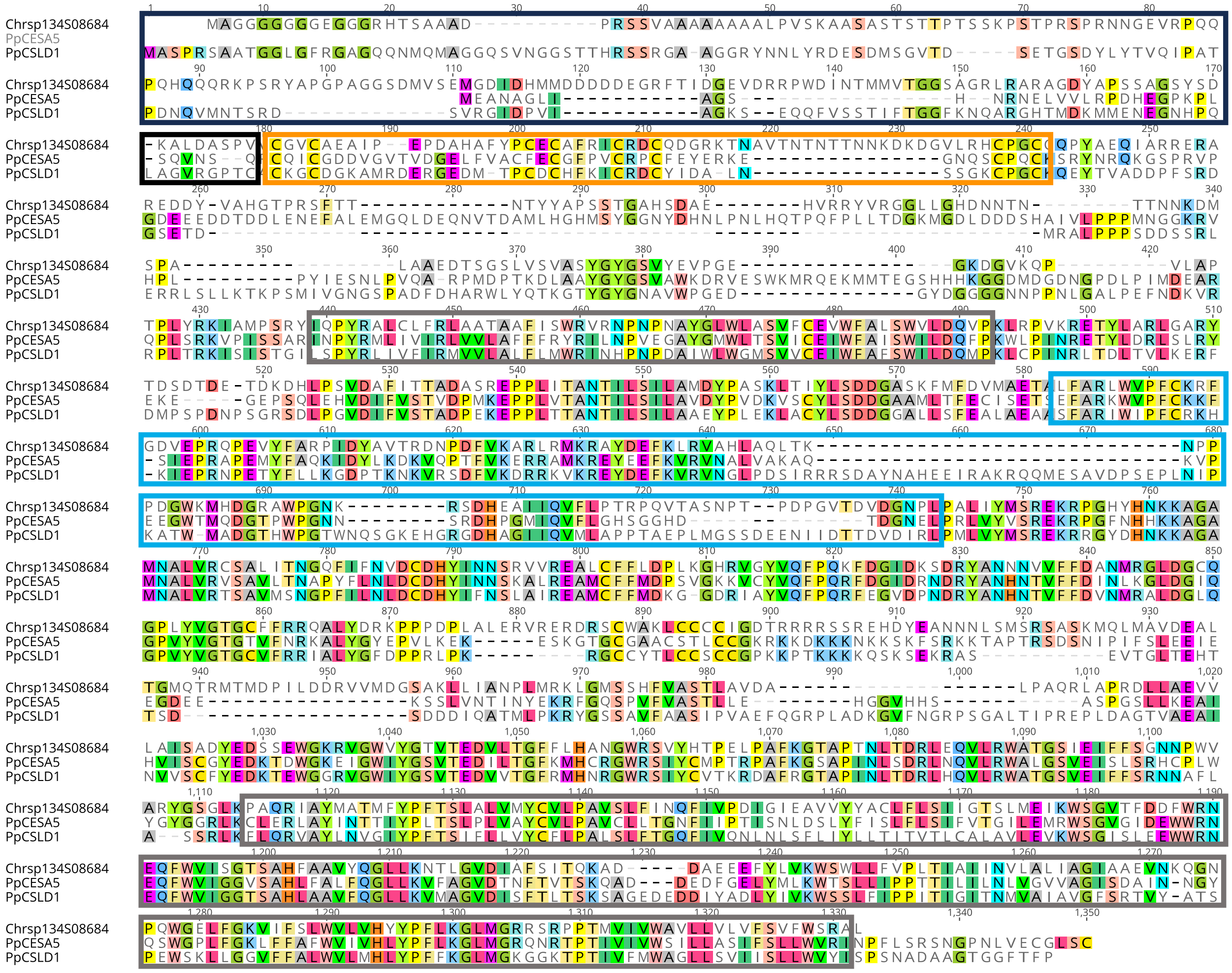

Fig. S8. Amino acid alignment of PpCSLD1, PpCESA5 and a CESA/CSLD-like sequence from *Chlorokybus atmophyticus*. The *C. atmophyticus* sequence (Chrsp134508684) (*46*) is more similar to PpCSLD1 in the N-terminus (black box) and RING-domain (orange box) and more similar to PpCESA5 in the plant-conserved region (cyan box). All three sequences are highly similar in the transmembrane regions (grey boxes). The alignment was constructed with CLUSTAL Omega (*70*).

Table S1. Oligonucleotides used for vector construction and genotype analysis.

Primer pair	Sequences	Amplicon size (bp)	Annealing temperature	Amplified region
CES5_gRna789r_s<br>CES5_gRna789r_a	ccatTCGAGACGTACACTAGCCTG<br>aaacCAGGCTAGTGTACGTCTCGA	NA	NA	Protospacer targeting *CESA5*
CES5_gRna1819r_s<br>CES5_gRna1819r_a	ccatCTACGGAACCCAAAGCCCAC<br>aaacGTGGGCTTTGGGTTCCGTAG	NA	NA	Protospacer targeting *CESA5*
CESA5_CRdel-F<br>CESA5_CRdel-R	CAACCGCGAGACATACTTGG<br>TGCTTGTTTGGAAGTGACGG	2146<br>1115	53°C	*CESA5* CRISPR target
CESA1_CRdel-F<br>CESA1_CRdel-R	GAAGCCTGTGAAAAGTCCGG<br>AGGCTGCACTCTATCCTTCA	3056<br>191	60°C	*CESA1* CRISPR target
CESA1gap-F<br>CESA1gap-R	CGGAAGGAGGTCAGCTTCAA<br>CGCAGATCACAGATGTCAGC	622	58°C	*CESA1* deletion test
CESA3gap-F<br>CESA3gap-R	TCAACAACAGCAAGGCCATC<br>TCTTACCCTTCTTGCGTCGT	457	58°C	*CESA3* deletion test
CESA4gap-F<br>CESA4gap-R	GGGTCGTATGGGTATGGGAG<br>GGTAGATCTGAGCAGTCCTGG	155	58°C	*CESA4* deletion test
CESA5gap-F<br>CESA5gap-R	TTCAACAGGAAGGCGCTCTA<br>CCGCAACTGATGACGTGAAT	377	58°C	*CESA5* deletion test
CESA8gap-F<br>CESA8gap-R	AAGAGGCCGGGATTCAATCA<br>CTCCTAAAGACACACCCCGT	456	58°C	*CESA8* deletion test
CESA10gap-F<br>CESA10gap-R	GCAATGCAGCCTACGTATCC<br>GGTGCCAATGTCGCTGTAAG	423	58°C	*CESA10* deletion test
CSLD3_CRdel-F<br>CSLD3_CRdel-R	CGACAAGGAGAAGGAGGAGG<br>GCGTGGTCAAAATCCGTAGT	791	58°C	DNA quality control
CESA6TargetF<br>CESA6TargetR	GTGAGGTGCGAGGAAGAAAG<br>TTCCCTAACTCCACCACTGC	142	60°C	*CESA6* deletion test
CESA7TargetF<br>CESA7TargetR	CTTGTGAGGAAGTGCGGGAA<br>ACATTACTCAACGGCCTCGG	1254	60°C	*CESA7* deletion test
CESA5_OffT_1F<br>CESA5_OffT_1R	GGTCAGGGTCACTTGGATCA<br>TTGTCGGCATGCTTTGGAAA	N.A.	51°C	Predicted off-target site 1
CESA5_OffT_2F<br>CESA5_OffT_2R	AAGACAGACTCGGGACAAGG<br>TCAACTGCCATTACTCTGCA	N.A.	51°C	Predicted off-target site 2
CESA5_OffT_3F<br>CESA5_OffT_3R	CAACGCAATGCAGTCTCAGA<br>AAGACATTCCAGGGGCAGC	N.A.	53°C	Predicted off-target site 3
CESA5_OffT_4F<br>CESA5_OffT_4R	ATGCGACAGGGGAGAGTATG<br>TTTCTCGTGGTGTTGCTGTG	N.A.	53°C	Predicted off-target site 4
CESA5_OffT_5F<br>CESA5_OffT_5R	CCAAGTGCCGGCAGTATTAC<br>GAGGACGTTGACAGTGGAGA	N.A.	54°C	Predicted off-target site 5
CESA3_CRdel-F<br>CESA3_CRdel-R	CCAAATGGCTCCCGATTCAG<br>CGTAGCCACAACTGATGACG	N.A.	60°C	*CESA3* CRISPR target
CESA8_CRdel-F<br>CESA8_CRdel-R	CCGTTTAGTGGTGTTGGCAT<br>GCAATGCCTACTGAGCGAAA	N.A.	60°C	*CESA8* CRISPR target
CESA4_CRdel-F2<br>CESA4_CRdel-R2	ACATCCCCAGATCATCAAGCT<br>GGGGCTCGATGTTGAACTT	N.A.	52°C	*CES4* CRISPR target
CESA10_CRdel-F<br>CESA10_CRdel-R	ACTCCGACGACCTAGACAAC<br>CTCCCTCTCCACTTGCTTGA	N.A.	54°C	*CES10* CRISPR target
C6KO_F2<br>CESA7KOFlankR2	GCTTCAATGCTGTACCACAAACCAC<br>AAGCCCTAACTTCCAGCACC	N.A.	55°C	*CES6/7* deletion

Supplementary auxiliary files:

Movie S1. Time lapse of gametophore bud development in CESA-deficient *P. patens*. Imaging commenced after several cell divisions when the developing rhizoid was approximately 50 μm long (time stamp in upper left corner = 00:00 hr:min). Cells ruptured at 5:20 hr:min and 8:50 hr:min and buds turned brown after the second cell rupture (9:00 hr:min). The time lapse interval = 10 min. See also Fig. 1.

Movie S2. Time lapse of gametophore bud development in CESA-deficient *P. patens*. Imaging commenced at the 4-cell stage (time stamp in upper left corner = 00:00 hr:min) and the developing rhizoid reached approximately 50 μm at 31:40 hr:min. Cell rupture occurred at 45:35 hr:min. The time lapse interval = 5 min.

REFERENCES AND NOTES

1. P. Purushotham, R. Ho, J. Zimmer, Architecture of a catalytically active homotrimeric plant cellulose synthase complex. *Science* **369**, 1089–1094 (2020).

2. B. T. Nixon, K. Mansouri, A. Singh, J. Du, J. K. Davis, J. G. Lee, E. Slabaugh, V. G. Vandavasi, H. O'Neill, E. M. Roberts, A. W. Roberts, Y. G. Yingling, C. H. Haigler, Comparative structural and computational analysis supports eighteen cellulose synthases in the plant cellulose synthesis complex. *Sci. Rep.* **6**, 28696 (2016).

3. P. A. Penttila, A. Paajanen, Critical comment on the assumptions leading to 24-chain microfibrils in wood. *Nat. Plants* **10**, 1064–1066 (2024).

4. D. Cosgrove, P. Dupree, E. D. Gomez, C. H. Haigler, J. D. Kubicki, J. Zimmer, How many glucan chains form plant cellulose microfibrils? A mini review *Biomacromolecules* **25**, 6357–6366 (2024).

5. H. C. Tai, C. S. Tsao, J. H. Lin, Reply to: Critical comment on the assumptions leading to 24-chain microfibrils in wood. *Nat. Plants* **10**, 1067–1070 (2024).

6. H.-C. Tai, C.-H. Chang, W. Cai, J.-H. Lin, S.-J. Huang, Q.-Y. Lin, E. C.-H. Yuan, S.-L. Li, Y.-J. Lin, J. C. C. Chan, C.-S. Tsao, Wood cellulose microfibrils have a 24-chain core-shell nanostructure in seed plants. *Nat. Plants* **9**, 1154–1168 (2023).

7. S. Persson, A. Paredez, A. Carroll, H. Palsdottir, M. Doblin, P. Poindexter, N. Khitrov, M. Auer, C. R. Somerville, Genetic evidence for three unique components in primary cell-wall cellulose synthase complexes in Arabidopsis. *Proc. Natl. Acad. Sci. U.S.A.* **104**, 15566–15571 (2007).

8. J. Yang, G. Bak, T. Burgin, W. J. Barnes, H. B. Mayes, M. J. Pena, B. R. Urbanowicz, E. Nielsen, Biochemical and genetic analysis identify CSLD3 as a beta-1,4-glucan synthase that functions during plant cell wall synthesis. *Plant Cell* **32**, 1749–1767 (2020).

9. R. G. Orr, X. Cheng, L. Vidali, M. Bezanilla, Orchestrating cell morphology from the inside out – using polarized cell expansion in plants as a model. *Curr. Opin. Cell Biol.* **62**, 46–53 (2019).

10. S. Z. Wu, A. M. Chaves, R. Li, A. W. Roberts, M. Bezanilla, Cellulose synthase-like D movement in the plasma membrane requires enzymatic activity. *J. Cell Biol.* **222**, (2023).

11. A. R. Paredez, C. R. Somerville, D. W. Ehrhardt, Visualization of cellulose synthase demonstrates functional association with microtubules. *Science* **312**, 1491–1495 (2006).

12. F. Diotallevi, B. Mulder, The cellulose synthase complex: A polymerization driven supramolecular motor. *Biophys. J.* **92**, 2666–2673 (2007).

13. P. B. Green, Mechanism for plant cellular morphogenesis. *Science* **138**, 1404–1405 (1962).

14. A. W. Roberts, J. T. Bushoven, The cellulose synthase (*CESA*) gene superfamily of the moss *Physcomitrella patens*. *Plant Mol. Biol.* **63**, 207–219 (2007).

15. X. Li, A. M. Chaves, D. C. T. Dees, N. Mansoori, K. Yuan, T. L. Speicher, J. H. Norris, I. Wallace, L. M. Trindade, A. W. Roberts, Cellulose synthesis complexes are homo-oligomeric and hetero-oligomeric in *Physcomitrella patens*. *Plant Physiol.* **188**, 2115–2130 (2022).

16. C. A. Goss, D. J. Brockmann, J. T. Bushoven, A. W. Roberts, A *CELLULOSE SYNTHASE* (*CESA*) gene essential for gametophore morphogenesis in the moss *Physcomitrella patens*. *Planta* **235**, 1355–1367 (2012).

17. C. J. Harrison, A. H. Roeder, E. M. Meyerowitz, J. A. Langdale, Local cues and asymmetric cell divisions underpin body plan transitions in the moss *Physcomitrella patens*. *Curr. Biol.* **19**, 461–471 (2009).

18. J. H. Norris, X. Li, S. Huang, A. M. L. Van de Meene, M. L. Tran, E. Killeavy, A. M. Chaves, B. Mallon, D. Mercure, H.-T. Tan, R. A. Burton, M. S. Doblin, S. H. Kim, A. W. Roberts, Functional specialization of cellulose synthase isoforms in a moss shows parallels with seed plants. *Plant Physiol.* **175**, 210–222 (2017).

19. D. Lang, K. K. Ullrich, F. Murat, J. Fuchs, J. Jenkins, F. B. Haas, M. Piednoel, H. Gundlach, M. Van Bel, R. Meyberg, C. Vives, J. Morata, A. Symeonidi, M. Hiss, W. Muchero, Y. Kamisugi, O. Saleh, G. Blanc, E. L. Decker, N. van Gessel, J. Grimwood, R. D. Hayes, S. W. Graham, L. E.

Gunter, S. F. McDaniel, S. N. W. Hoernstein, A. Larsson, F. W. Li, P. F. Perroud, J. Phillips, P. Ranjan, D. S. Rokshar, C. J. Rothfels, L. Schneider, S. Shu, D. W. Stevenson, F. Thummler, M. Tillich, J. C. Villarreal Aguilar, T. Widiez, G. K. Wong, A. Wymore, Y. Zhang, A. D. Zimmer, R. S. Quatrano, K. F. X. Mayer, D. Goodstein, J. M. Casacuberta, K. Vandepoele, R. Reski, A. C. Cuming, G. A. Tuskan, F. Maumus, J. Salse, J. Schmutz, S. A. Rensing, The *Physcomitrella patens* chromosome-scale assembly reveals moss genome structure and evolution. *Plant J.* **93**, 515–533 (2018).

20. G. Bi, S. Zhao, J. Yao, H. Wang, M. Zhao, Y. Sun, X. Hou, F. B. Haas, D. Varshney, M. Prigge, S. A. Rensing, Y. Jiao, Y. Ma, J. Yan, J. Dai, Near telomere-to-telomere genome of the model plant Physcomitrium patens. *Nat. Plants* **10**, 327–343 (2024).

21. C. T. Anderson, A. Carroll, L. Akhmetova, C. Somerville, Real-time imaging of cellulose reorientation during cell wall expansion in Arabidopsis roots. *Plant Physiol.* **152**, 787–796 (2010).

22. R. J. Vietor, R. H. Newman, M. A. Ha, D. C. Apperley, M. C. Jarvis, Conformational features of crystal-surface cellulose from higher plants. *Plant J.* **30**, 721–731 (2002).

23. M.-A. Ha, D. C. Apperley, B. W. Evans, I. M. Huxham, W. G. Jardine, R. J. Viëtor, D. Reis, B. Vian, M. C. Jarvis, Fine structure in cellulose microfibrils: NMR evidence from onion and quince. *Plant J.* **16**, 183–190 (1998).

24. R. H. Newman, Evidence for assignment of ^{13}C NMR signals to cellulose crystallite surfaces in wood, pulp and isolated celluloses. *Holzforschung* **52**, 157–159 (1998).

25. R. M. Brown Jr., D. Montezinos, Cellulose microfibrils: Visualization of biosynthetic and orienting complexes in association with the plasma membrane. *Proc. Natl. Acad. Sci. U.S.A.* **73**, 143–147 (1976).

26. S. C. Mueller, R. M. Brown, Jr., Evidence for an intramembrane component associated with a cellulose microfibril-synthesizing complex in higher plants. *J. Cell Biol.* **84**, 315–326 (1980).

27. I. Tsekos, The sites of cellulose synthesis in algae: Diversity and evolution of cellulose-synthesizing enzyme complexes. *J. Phycol.* **35**, 635–655 (1999).

28. S. Kimura, W. Laosinchai, T. Itoh, X. Cui, C. R. Linder, R. M. Brown Jr., Immunogold labeling of rosette terminal cellulose-synthesizing complexes in the vascular plant *Vigna angularis*. *Plant Cell* **11**, 2075–2085 (1999).

29. T. H. Giddings Jr., D. L. Brower, L. A. Staehelin, Visualization of particle complexes in the plasma membrane of *Micrasterias denticulata* associated with the formation of cellulose fibrils in primary and secondary cell walls. *J. Cell Biol.* **84**, 327–339 (1980).

30. N. J. Severs, Freeze-fracture electron microscopy. *Nat. Protoc.* **2**, 547–576 (2007).

31. A. W. Roberts, E. M. Roberts, C. H. Haigler, Moss cell walls: Structure and biosynthesis. *Front. Plant Sci.* **3**, 166 (2012).

32. Y. Watanabe, R. Schneider, S. Barkwill, E. Gonzales-Vigil, J. L. Hill Jr., A. L. Samuels, S. Persson, S. D. Mansfield, Cellulose synthase complexes display distinct dynamic behaviors during xylem transdifferentiation. *Proc. Natl. Acad. Sci. U.S.A.* **115**, E6366–E6374 (2018).

33. C. H. Haigler, R. M. Brown, Jr., Transport of rosettes from the Golgi apparatus to the plasma membrane in isolated mesophyll cells of *Zinnia elegans* during differentiation to tracheary elements in suspension culture. *Protoplasma* **134**, 111–120 (1986).

34. N. G. Taylor, R. M. Howells, A. K. Huttly, K. Vickers, S. R. Turner, Interactions among three distinct CesA proteins essential for cellulose synthesis. *Proc. Natl. Acad. Sci. U.S.A.* **100**, 1450–1455 (2003).

35. B. M. Kiedaisch, R. L. Blanton, C. H. Haigler, Characterization of a novel cellulose synthesis inhibitor. *Planta* **217**, 922–930 (2003).

36. G. Tang, L. Peng, P. R. Baldwin, D. S. Mann, W. Jiang, I. Rees, S. J. Ludtke, EMAN2: An extensible image processing suite for electron microscopy. *J. Struct. Biol.* **157**, 38–46 (2007).

37. T. Beeckman, G. K. Przemeck, G. Stamatiou, R. Lau, N. Terryn, R. De Rycke, D. Inze, T. Berleth, Genetic complexity of cellulose synthase a gene function in Arabidopsis embryogenesis. *Plant Physiol.* **130**, 1883–1893 (2002).

38. C. S. Gillmor, P. Poindexter, J. Lorieu, M. M. Palcic, C. Somerville, α-glycosidase I is required for cellulose biosynthesis and morphogenesis in *Arabidopsis*. *J. Cell Biol.* **156**, 1003–1013 (2002).

39. B. Favery, E. Ryan, J. Foreman, P. Linstead, K. Boudonck, M. Steer, P. Shaw, L. Dolan, *KOJAK* encodes a cellulose synthase-like protein required for root hair cell morphogenesis in *Arabidopsis*. *Genes Dev.* **15**, 79–89 (2001).

40. W. Wang, L. Wang, C. Chen, G. Xiong, X. Y. Tan, K. Z. Yang, Z. C. Wang, Y. Zhou, D. Ye, L. Q. Chen, Arabidopsis CSLD1 and CSLD4 are required for cellulose deposition and normal growth of pollen tubes. *J. Exp. Bot.* **62**, 5161–5177 (2011).

41. A. H. Liepman, C. J. Nairn, W. G. T. Willats, I. Sørensen, A. W. Roberts, K. Keegstra, Functional genomic analysis supports conservation of function among *Cellulose synthase-like A* gene family members and suggests diverse roles of mannans in plants. *Plant Physiol.* **143**, 1881–1893 (2007).

42. J. Davis, F. Brandizzi, A. H. Liepman, K. Keegstra, Arabidopsis mannan synthase CSLA9 and glucan synthase CSLC4 have opposite orientations in the Golgi membrane. *Plant J.* **64**, 1028–1037 (2010).

43. I. Kurek, Y. Kawagoe, D. Jacob-Wilk, M. Doblin, D. Delmer, Dimerization of cotton fiber cellulose synthase catalytic subunits occurs via oxidation of the zinc-binding domains. *Proc. Natl. Acad. Sci. U.S.A.* **99**, 11109–11114 (2002).

44. T. A. Richmond, C. R. Somerville, The cellulose synthase superfamily. *Plant Physiol.* **124**, 495–498 (2000).

45. E. Fitzek, L. Orton, S. Entwistle, W. S. Grayburn, C. Ausland, M. R. Duvall, Y. Yin, Cell wall enzymes in *Zygnema circumcarinatum* UTEX 1559 respond to osmotic stress in a plant-like fashion. *Front. Plant Sci.* **10**, 732 (2019).

46. S. Wang, L. Li, H. Li, S. K. Sahu, H. Wang, Y. Xu, W. Xian, B. Song, H. Liang, S. Cheng, Y. Chang, Y. Song, Z. Cebi, S. Wittek, T. Reder, M. Peterson, H. Yang, J. Wang, B. Melkonian, Y. Van de Peer, X. Xu, G. K. Wong, M. Melkonian, H. Liu, X. Liu, Genomes of early-diverging streptophyte algae shed light on plant terrestrialization. *Nat. Plants* **6**, 95–106 (2020).

47. X. Feng, J. Zheng, I. Irisarri, H. Yu, B. Zheng, Z. Ali, S. de Vries, J. Keller, J. M. R. Furst-Jansen, A. Dadras, J. M. S. Zegers, T. P. Rieseberg, A. Dhabalia Ashok, T. Darienko, M. J. Bierenbroodspot, L. Gramzow, R. Petroll, F. B. Haas, N. Fernandez-Pozo, O. Nousias, T. Li, E. Fitzek, W. S. Grayburn, N. Rittmeier, C. Permann, F. Rumpler, J. M. Archibald, G. Theissen, J. P. Mower, M. Lorenz, H. Buschmann, K. von Schwartzenberg, L. Boston, R. D. Hayes, C. Daum, K. Barry, I. V. Grigoriev, X. Wang, F. W. Li, S. A. Rensing, J. Ben Ari, N. Keren, A. Mosquna, A. Holzinger, P. M. Delaux, C. Zhang, J. Huang, M. Mutwil, J. de Vries, Y. Yin, Genomes of multicellular algal sisters to land plants illuminate signaling network evolution. *Nat. Genet.* **56**, 1018–1031 (2024).

48. M. D. Mikkelsen, J. Harholt, P. Ulvskov, I. E. Johansen, J. U. Fangel, M. S. Doblin, A. Bacic, W. G. T. Willats, Evidence for land plant cell wall biosynthetic mechanisms in charophyte green algae. *Ann. Bot.* **114**, 1217–1236 (2014).

49. Y. Yin, M. A. Johns, H. Cao, M. Rupani, A survey of plant and algal genomes and transcriptomes reveals new insights into the evolution and function of the cellulose synthase superfamily. *BMC Genomics* **15**, 260 (2014).

50. T. H. Wilson, M. Kumar, S. R. Turner, The molecular basis of plant cellulose synthase complex organisation and assembly. *Biochem. Soc. Trans.* **49**, 379–391 (2021).

51. G. B. Pedersen, L. Blaschek, K. E. H. Frandsen, L. C. Noack, S. Persson, Cellulose synthesis in land plants. *Mol. Plant* **16**, 206–231 (2023).

52. B. J. Harris, J. W. Clark, D. Schrempf, G. J. Szollosi, P. C. J. Donoghue, A. M. Hetherington, T. A. Williams, Divergent evolutionary trajectories of bryophytes and tracheophytes from a complex common ancestor of land plants. *Nat. Ecol. Evol.* **6**, 1634–1643 (2022).

53. F. Gu, M. Bringmann, J. R. Combs, J. Yang, D. C. Bergmann, E. Nielsen, Arabidopsis CSLD5 functions in cell plate formation in a cell cycle-dependent manner. *Plant Cell* **28**, 1722–1737 (2016).

54. D. Delmer, R. A. Dixon, K. Keegstra, D. Mohnen, The plant cell wall-dynamic, strong, and adaptable-is a natural shapeshifter. *Plant Cell* **36**, 1257–1311 (2024).

55. P. K. Hepler, Calcium: A central regulator of plant growth and development. *Plant Cell* **17**, 2142–2155 (2005).

56. S. Li, L. Lei, C. R. Somerville, Y. Gu, Cellulose synthase interactive protein 1 (CSI1) links microtubules and cellulose synthase complexes. *Proc. Natl. Acad. Sci. U.S.A.* **109**, 185–190 (2012).

57. M. Ogden, S. J. Whitcomb, G. A. Khan, U. Roessner, R. Hoefgen, S. Persson, Cellulose biosynthesis inhibitor isoxaben causes nutrient-dependent and tissue-specific Arabidopsis phenotypes. *Plant Physiol.* **194**, 612–617 (2024).

58. A. W. Roberts, C. S. Dimos, M. J. Budziszek, Jr., C. A. Goss, V. Lai, A. M. Chaves, Knocking out the wall: Revised protocols for gene targeting in *Physcomitrella patens*. *Methods Mol. Biol.* **2149**, 125–144 (2020).

59. D. R. Mallett, M. Chang, X. Cheng, M. Bezanilla, Efficient and modular CRISPR-Cas9 vector system for *Physcomitrella patens*. *Plant Direct* **3**, e00168 (2019).

60. M. Haeussler, K. Schonig, H. Eckert, A. Eschstruth, J. Mianne, J. B. Renaud, S. Schneider-Maunoury, A. Shkumatava, L. Teboul, J. Kent, J. S. Joly, J. P. Concordet, Evaluation of off-target and on-target scoring algorithms and integration into the guide RNA selection tool CRISPOR. *Genome Biol.* **17**, 148 (2016).

61. C. S. Bascom, Jr., S. Z. Wu, K. Nelson, J. Oakey, M. Bezanilla, Long-term growth of moss in microfluidic devices enables subcellular studies in development. *Plant Physiol.* **172**, 28–37 (2016).

62. D. M. Updegraff, Semimicro determination of cellulose in biological materials. *Anal. Biochem.* **32**, 420–424 (1969).

63. B. M. Fung, A. K. Khitrin, K. Ermolaev, An improved broadband decoupling sequence for liquid crystals and solids. *J. Magn. Reson.* **142**, 97–101 (2000).

64. H. Fukuda, A. Komamine, Establishment of an experimental system for the study of tracheary element differentiation from single cells isolated from the mesophyll of *Zinnia elegans*. *Plant Physiol.* **65**, 57–60 (1980).

65. D. Branton, S. Bullivant, N. B. Gilula, M. J. Karnovsky, H. Moor, K. Muhlethaler, D. H. Northcote, L. Packer, B. Satir, P. Satir, V. Speth, L. A. Staehlin, R. L. Steere, R. S. Weinstein, Freeze-etching nomenclature. *Science* **190**, 54–56 (1975).

66. C. H. Haigler, M. J. Grimson, J. Gervais, N. Le Moigne, H. Hofte, B. Monasse, P. Navard, Molecular modeling and imaging of initial stages of cellulose fibril assembly: Evidence for a disordered intermediate stage. *PLOS ONE* **9**, e93981 (2014).

67. J. Schindelin, I. Arganda-Carreras, E. Frise, V. Kaynig, M. Longair, T. Pietzsch, S. Preibisch, C. Rueden, S. Saalfeld, B. Schmid, J. Y. Tinevez, D. J. White, V. Hartenstein, K. Eliceiri, P. Tomancak, A. Cardona, Fiji: An open-source platform for biological-image analysis. *Nat. Methods* **9**, 676–682 (2012).

68. L. Li, S. Wang, H. Wang, S. K. Sahu, B. Marin, H. Li, Y. Xu, H. Liang, Z. Li, S. Cheng, T. Reder, Z. Cebi, S. Wittek, M. Petersen, B. Melkonian, H. Du, H. Yang, J. Wang, G. K. Wong, X. Xu, X. Liu, Y. Van de Peer, M. Melkonian, H. Liu, The genome of *Prasinoderma coloniale* unveils the existence of a third phylum within green plants. *Nat. Ecol. Evol.* **4**, 1220–1231 (2020).

69. A. W. Roberts, E. Roberts, Cellulose synthase (*CesA*) genes in algae and seedless plants. *Cellul.* **11**, 419–435 (2004).

70. F. Madeira, M. Pearce, A. R. N. Tivey, P. Basutkar, J. Lee, O. Edbali, N. Madhusoodanan, A. Kolesnikov, R. Lopez, Search and sequence analysis tools services from EMBL-EBI in 2022. *Nucleic Acids Res.* **50**, W276–W279 (2022).
